# A bispecific CAR-T cell therapy targeting BCMA and CD38 in relapsed or refractory multiple myeloma

**DOI:** 10.1186/s13045-021-01170-7

**Published:** 2021-10-09

**Authors:** Heng Mei, Chenggong Li, Huiwen Jiang, Xinying Zhao, Zhiping Huang, Dan Jin, Tao Guo, Haiming Kou, Lin Liu, Lu Tang, Ping Yin, Zhihui Wang, Lisha Ai, Sha Ke, Yimeng Xia, Jun Deng, Lei Chen, Li Cai, Chunyan Sun, Linghui Xia, Gaoquan Hua, Yu Hu

**Affiliations:** 1 Institute of Hematology, Union Hospital, Tongji Medical College, Huazhong University of Science and Technology, Wuhan, 430030 China; 2 Hubei Clinical Medical Center of Cell Therapy for Neoplastic Disease, Wuhan, 430022 China; 3 Institute of Hematology, Jingzhou Central Hospital, The Second Clinical Medical College, Yangtze University, Jingzhou, 434020 China; 4 Zhejiang Cellyan Biotechnology Co. Ltd, Jiaxin, 314001 China; 5 Department of Epidemiology and Biostatistics, School of Public Health, Tongji Medical College, Huazhong University of Science and Technology, Wuhan, 430030 China; 6 Drug Clinical Trial Institution, Union Hospital, Tongji Medical College, Huazhong University of Science and Technology, Wuhan, 430022 China

**Keywords:** Chimeric antigen receptor-T cells, Multiple myeloma, BCMA, CD38, Bispecific CAR

## Abstract

**Background:**

BCMA-specific chimeric antigen receptor-T cells (CAR-Ts) have exhibited remarkable efficacy in refractory or relapsed multiple myeloma (RRMM); however, primary resistance and relapse exist with single-target immunotherapy. Bispecific CARs are proposed to mitigate these limitations.

**Methods:**

We constructed a humanized bispecific BM38 CAR targeting BCMA and CD38 and tested the antimyeloma activity of BM38 CAR-Ts in vitro and in vivo. Twenty-three patients with RRMM received infusions of BM38 CAR-Ts in a phase I trial.

**Results:**

BM38 CAR-Ts showed stronger in vitro cytotoxicity to heterogeneous MM cells than did T cells expressing an individual BCMA or CD38 CAR. BM38 CAR-Ts also exhibited potent antimyeloma activity in xenograft mouse models. In the phase I trial, cytokine release syndrome occurred in 20 patients (87%) and was mostly grade 1–2 (65%). Neurotoxicity was not observed. Hematologic toxicities were common, including neutropenia in 96% of the patients, leukopenia in 87%, anemia in 43% and thrombocytopenia in 61%. At a median follow-up of 9.0 months (range 0.5 to 18.5), 20 patients (87%) attained a clinical response and minimal residual disease-negativity (≤ 10^–4^ nucleated cells), with 12 (52%) achieving a stringent complete response. Extramedullary plasmacytoma was eliminated completely in 56% and partially in 33% and of 9 patients. The median progression-free survival was 17.2 months. Two relapsed patients maintained BCMA and CD38 expression on MM cells. Notably, BM38 CAR-Ts cells were detectable in 77.8% of evaluable patients at 9 months and 62.2% at 12 months.

**Conclusion:**

Bispecific BM38 CAR-Ts were feasible, safe and significantly effective in patient with RRMM.

*Trial registration*: Chictr.org.cn ChiCTR1800018143.

**Supplementary Information:**

The online version contains supplementary material available at 10.1186/s13045-021-01170-7.

## Background

Multiple myeloma (MM) represents one of the most common hematological malignancies with substantial morbidity and mortality [1, 2]. Several emerging agents have improved the prognosis of patients with MM in recent years, with the 5-year overall survival (OS) rate rising from ~ 30 to ~ 60% [3–5]. Nevertheless, MM remains an incurable disease, and patients with relapsed or refractory (RR) disease tend to have poor outcomes [6]. Among patients with high-risk profiles, including unfavorable cytogenetic abnormalities and extramedullary disease (EMD), the survival is worse [7, 8]. Novel therapeutic strategies are needed for these individuals.

Chimeric antigen receptor-T cells (CAR-Ts) have shown promising results in the treatment of RRMM. CAR targets, including B-cell maturation antigen (BCMA) [9–16], CD19 [17, 18], CS1 (SLAMF7) [19, 20], CD38 [21–23], GPRC5D [24], kappa light chain [25] and NKG2DL [26], have been actively examined. BCMA expression is restrictive to normal and malignant plasma cells and some B cells [27], and is consequently regarded as an ideal target in MM. BCMA-directed CAR-Ts have achieved striking clinical response rates (81–97%) in patients with RRMM [9–15], but relapse occurs in approximately 45% of responders [28]. BCMA downregulation or loss is observed in 4–33% of progressive patients post-anti-BCMA CAR-T cell therapy [10, 12, 29]. T cells expressing bispecific CARs have previously found to be an effective strategy to mitigate the limitations and enhance functionality [30–35]. One patient relapsed after BCMA-targeted immunotherapy with irreversible BCMA loss, but CD38 expression was enhanced on MM cells [36]. Prompted by this observation, we designed a second-generation bispecific BM38 CAR containing a fully human anti-BCMA single-chain variable fragment (scFv) and a humanized anti-CD38 scFv.

CD38 is extensively and highly expressed on MM cells, including therapy-resistant myeloma-initiating cells [37, 38]. CD38-targeted CAR-Ts have displayed anti-MM activity in preclinical studies [21–23]. The major concern regarding CD38 as a CAR target is its weak expression on hematopoietic progenitor cells (HPCs) and normal T cells [39], hence facing potential risks of myelotoxicity and fratricide. A rational strategy by affinity optimization has been established to diminish the undesired on-target off-tumor effects, and T cells expressing CD38-directed CARA4, which had reduced affinity (K_D_ 10^−7^ M to 10^−5^ M), could selectively eliminate CD38^++^ MM cells, but spare CD38^+^ HPCs and T cells [40]. Therefore, we lowered the affinity of anti-CD38 scFv (K_D_ 5.2 × 10^−7^ M to 4.2 × 10^−5^ M) in the BM38 CAR and maintained the high affinity of anti-BCMA scFv (K_D_ < 5.4 × 10^−8^ M) for major cytotoxicity. Herein, we report the preclinical results of the bispecific BM38 CAR-Ts and the outcomes of the patients with RRMM treated in a phase I trial.

## Methods

### Study design

We conducted a preclinical assessment, including evaluation of transduction efficiency, phenotype, in vitro proliferation, cytotoxicity and cytokine production, of T cells modified to express bispecific BM38 CARs, 38BM CARs, and individual BCMA CARs and CD38 CARs. BCMA, 38BM and BM38 CARs with stronger in vitro antitumor activity were further evaluated in xenogeneic mouse models. Next, we performed a phase I clinical trial of BM38 CAR-Ts in patients with RRMM.

### Cell lines

The MM cell lines MM.1s (BFN60808555), U266 (BFN60800676) and RPMI-8226 (BFN608006107) were purchased from the Shanghai Cell Center-American Type Culture Collection (Shanghai, China). Raji (GDC191), Jurkat (GDC094) and K562 (GDC037) cells were purchased from the China Center for Type Culture Collection (Wuhan, China). K562 cells were transfected to express human BCMA (K562-BCMA) with lentiviral vectors in the authors’ laboratory. All the cell lines were authenticated for CD38 (anti-CD38-APC, 555335; BD Biosciences [BD], USA) and BCMA (anti-BCMA-PE, 130-119-147; Miltenyi Biotec, USA) expression by flow cytometry, and cultured in RPMI 1640 medium (Gibco, USA) containing 10% fetal bovine serum (NQBB, China).

### CAR construction

**Fig. 1:**
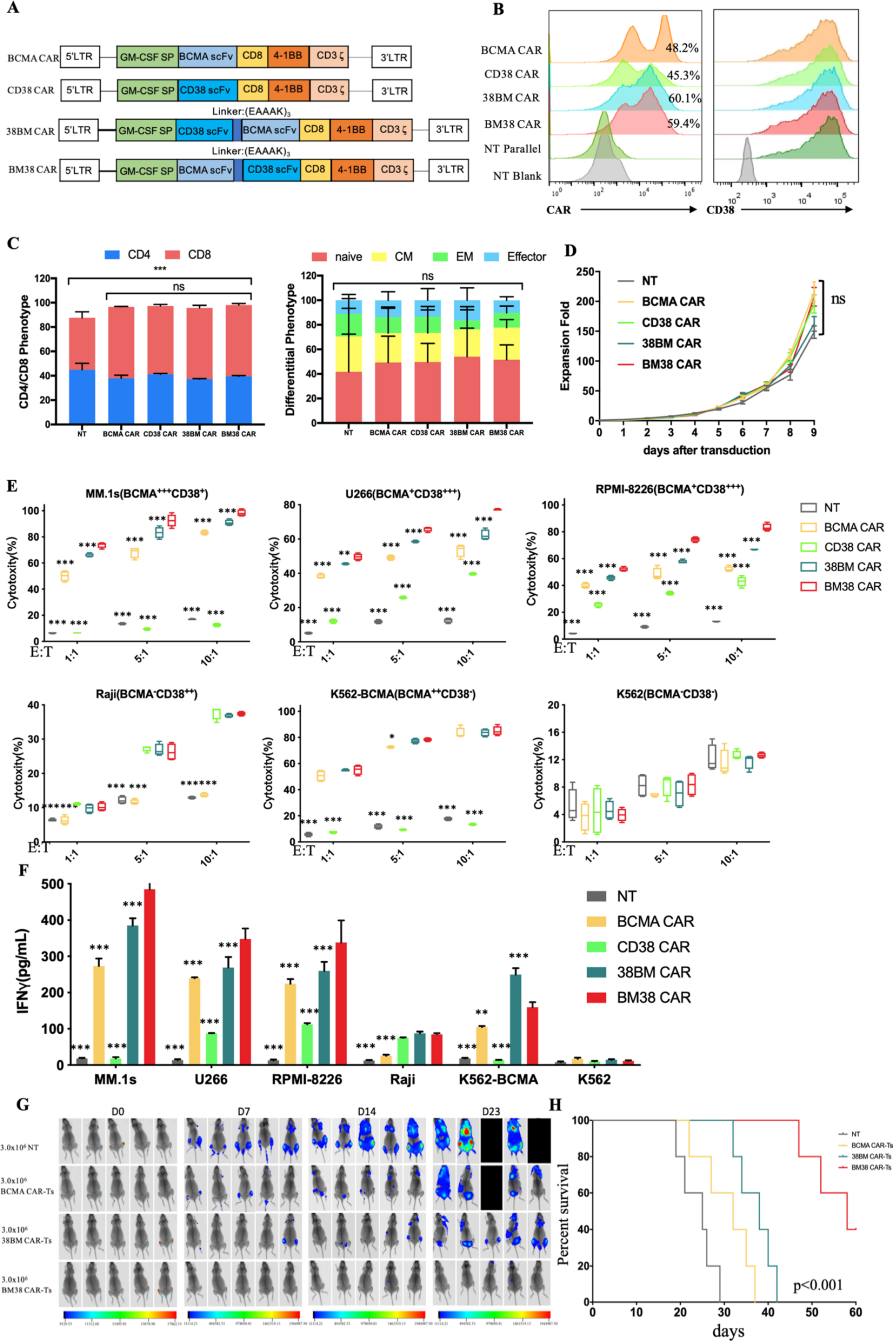
Preclinical evaluation of BM38 CAR-Ts. **a** Schematic structure of single-target BCMA CAR, CD38 CAR, bispecific 38BM CAR and BM38 CAR. All the CARs contained a granulocyte–macrophage colony-stimulating factor signal peptide (GM-CSF SP), a CD8 hinge and transmembrane domain, a 4-1BB costimulatory domain and a CD3ζ signaling domain. **b** CAR expression on lentivirus-transduced T cells and CD38 expression on CAR-Ts. Histograms are representative of 3 independent experiments. NT, nontransduced T. NT blank, NT without antibody staining. NT parallel, NT with antibody staining. **c** Phenotypic profiles of the final CAR-T products and NT cells. Differentiation phenotypes are depicted for naïve (CD45RA^+^CD62L^+^), central memory (CM) (CD45RA^−^CD62L^+^), effector memory (EM) (CD45RA^−^CD62L^−^) and effector (CD45RA^+^CD62L^−^) cells. *n* = 3, mean ± standard error of mean (SEM). Statistical analysis was performed using one-way ANOVA and subsequent multiple comparisons with BM38 CAR-Ts. ***P < 0.001; ns: not significant. **d** Expansion curves of the four CAR-Ts and NT cells after lentiviral transduction. *n* = 5, mean ± SEM. Repeated measures one-way ANOVA was used, ns: not significant. **e** Cytotoxicity of the four CAR-Ts and NT cells to target cells at an effector: target (E:T) ratio of 1:1, 5:1 and 10:1. *n* = 4, mean ± SEM. Dunnett’s multiple comparisons test was used at every E:T ratio with BM38 CAR-Ts as the control. *P < 0.05; **P < 0.01; ***P < 0.001; ns: not significant. **f** Production of interferon γ (IFNγ) at an E:T ratio of 1:1. *n* = 3, mean ± SEM. Dunnett’s multiple comparisons test was used with BM38 CAR-Ts as control. **P < 0.01; ***P < 0.001; ns: not significant. **g** Luciferase live imaging of MM.1s xenograft mice on day 0, 7, 14 and 23 after infusion of 3.0 × 10^6^ NT cells, BCMA CAR-Ts, 38BM CAR-Ts or BM38 CAR-Ts. **h** Kaplan–Meier survival plot of MM xenograft mice. The log-rank test was used

Sequences of the fully human anti-BCMA scFv and the humanized anti-CD38 scFv were obtained from the patent (CN 109503716A). The fully human anti-BCMA scFv was selected from Human Phage Display Libraries. The humanization of the anti-CD38 scFv consisted of replacement of the framework region and grafting of the complementary-determining region with human antibody fragments. In view of previous studies,^16,22^ the affinity of anti-CD38 scFv was reduced from 5.2 × 10^−7^ to 4.2 × 10^−5^ M to diminish undesirable on-target off-tumor effects. The affinity of the anti-BCMA scFv was maintained at < 5.4 × 10^−8^ M to keep strong cytotoxicity, and the affinities were verified by ELISA. The bispecific BM38 and 38BM CARs contained the anti-BCMA scFv and anti-CD38 scFv in tandem, linked by (EAAAK)_3_. A schematic CAR structure is provided in Fig. 1a. The CAR sequences were ligated into the lentiviral backbone plasmid (pLVX-EF1) and confirmed by gene sequencing (Anoroad, China).

### Virus production and titration

Lentiviruses were generated by plating 293 T packaging cells (CRL-3216, ATCC, USA) transfected with the following plasmids (Addgene, USA): pRSV.REV encoding rev, pMDLg/p.RRE encoding gag and pol, pVSV-G encoding the vesicular stomatitis virus envelope protein and pLVX-EF1 encoding the desired CAR. The lentiviral supernatants were collected after 72 h, concentrated via ultracentrifugation and PEF filtration and stored at − 80 °C.

### Manufacturing of BM38 CAR-Ts

After informed consent was obtained, at least 1.2 × 10^9^ peripheral blood mononuclear cells (PBMCs) were collected from enrolled patients by leukapheresis (COBE Spectra, USA). PBMCs were isolated by Ficoll density centrifugation (Sigma-Aldrich, Germany) and activated with an anti-CD3 antibody and anti-CD28 antibody (T&L Biotechnology, China) for 24 h. At least 1.6 × 10^7^ activated T cells were suspended in lentiviral supernatants at a multiplicity of infection of 5 for 6 h and then expanded ex vivo for 9–11 days in the presence of recombinant human interleukin (IL)-2 (30 U/mL, Huaxin Biotechnology, China). The expected number of CAR-Ts was prepared for infusion if the predefined release criteria, including transduction efficiency ≥ 10%, cell viability ≥ 70%, mycoplasma negativity, bacterial and fungal culture negativity, were passed.

### Proliferation and phenotype identification

Approximately 2.0 × 10^7^ T cells were transduced with lentivirus, and trypan blue staining was used to count live cells every 24 h to draw cellular growth curves. BM38, 38BM and BCMA CAR expression was detected using biotinylated human BCMA (BC7-H82FO; ACRO Biosystems, USA), while CD38 CAR expression was detected using protein L (A033-1901; GeneScript, China), and all the samples were then stained with streptavidin-PE (405203; BioLegend, USA). Anti-CD3-APC (561811; BD, USA), anti-CD4-BB515(564419; BD), anti-CD8-PerCP (344708; BioLegend), anti-CD62L-FITC (555543; BD), anti-CD45RA-PerCP-Cy5.5(563,429; BD), anti-CD3-FITC (561806; BD) and anti-CD38-APC (555335; BD) were used for phenotype identification of the final CAR-T cell products. Data were collected using CytoFLEX S flow cytometer (Beckman, USA) and analyzed using FlowJo software (TreeStar, USA).

### Cytotoxicity assay

Target cells (MM.1s, U266, RPMI-8226, Raji, K562 or K562-BCMA) were seeded at 3 × 10^4^ cells per well in 96-well plates and coincubated with effector cells (BM38 CAR-Ts, 38BM CAR-Ts, BCMA CAR-Ts, CD38 CAR-Ts or nontransduced T (NT) cells) at an effector-to-target (E:T) ratio of 1:1, 5:1 or 10:1 for 16 h in quadruplicate. The corresponding numbers of target cells and effector cells were cultured separately as controls for natural release. The supernatants were harvested and analyzed using the LDH Cytotoxicity Assay Kit (C0016; Beyotime Biotech, China) according to the manufacturer’s instructions. OD values were obtained at 492 nm by MB530 Microplate Reader (Huisong, China). Cytotoxicity was calculated as follows: cytotoxicity (%) = (OD values of coincubated cells—OD values of natural release of target cells—OD values of natural release of effector cells) / (OD values of maximum release of target cells—OD values of natural release of target cells) × 100%.

### Cytokine detection

Target cells (MM.1s, U266, RPMI-8226, Raji, K562 or K562-BCMA cells) were seeded at 2 × 10^4^ cells per well in 96-well plates and coincubated with effector cells (BM38 CAR-Ts, 38BM CAR-Ts, BCMA CAR-Ts, CD38 CAR-Ts or NT cells) at an E:T ratio of 1:1 for 16 h in triplicate. The supernatants were harvested to assess the levels of interferon *γ* (560111; BD) according to the manufacturer’s instructions.

### MM xenograft model

MM xenograft mouse models were established with 12-week-old male NOD-Prkdcscid IL2rgtm/Bcgen (B-NDG) mice (Biocytogen, China) by tail vein injection of 1 × 10^6^ luciferase-expressing MM.1s cells per mouse on day −1. In view of the in vitro cytotoxicity of CAR-Ts to MM.1s cells, CD38 CAR-Ts were not further tested in vivo. Mice were randomly divided into 4 groups (*n* = 5) and injected with a single dose of 3.0 × 10^6^ NT cells, BCMA CAR-Ts, 38BM CAR-Ts or BM38 CAR-Ts on day 0. In vivo tumor growth was evaluated by luciferase live imaging (Bruker Daltonics, Germany) on day 0, 7, 14 and 23. The mice were observed for mortality up to 60 days after modeling. Survival is graphically represented as Kaplan–Meier curves and was analyzed by using the log-rank test.

### Clinical trial design

This open-label, single-arm, single-center, phase 1 dose-climbing and expansion study was approved by the Ethics Committee of the Union Hospital affiliated to Huazhong University of Science and Technology, Wuhan, China (REC ref no. [2018] 005). The study was performed in accordance with the principles of the Declaration of Helsinki and the International Conference on Harmonisation Guidelines for Good Clinical Practice. All patients provided written informed consent. This study was registered on Chictr.org.cn, number ChiCTR1800018143.

Eligible subjects were aged 18 years or older, had histopathologically confirmed MM and met the diagnostic criteria for RRMM defined by the International Myeloma Working Group (IMWG) [41]. Patients had experienced relapse or were refractory to at least 2 prior lines of therapy, including a proteasome inhibitor and an immunomodulatory drug. Measurable disease and good organ function were required. Positive BCMA or CD38 expression on MM cells was required to be confirmed by immunohistochemistry (IHC) or flow cytometry, but no prespecified level of expression was required. An Eastern Cooperative Oncology Group (ECOG) performance status greater than 2 caused by osteolytic destruction of MM was accepted.

Patients received lymphodepleting regimens consisting of cyclophosphamide (250 mg/m^2^, day − 5 to − 3) and fludarabine (25 mg/m^2^, day − 5 to − 3). The dose gradients were 0.5, 1.0, 2.0, 3.0 and 4.0 × 10^6^ CAR-Ts/kg. A modified 3 + 3 clinical design was used. Dose-limiting toxicity was observed within 28 days post-infusion and defined as hypotension refractory to high-dose vasopressors, or hypoxia requiring mechanical ventilation, or death attributed to CAR-T cell infusion. In view of the previous results of LCAR-B38M [13], a dual-epitope CAR for RRMM, and our early observation of patient 1–4, the dose of 0.5 × 10^6^ and 1.0 × 10^6^ CAR-Ts/kg was considered sufficiently safe, and therefore only 2 patients were enrolled in the two groups for risk–benefit trade-off. Responses were assessed on day 14 ± 2 and 28 ± 2 after infusion, and at each follow-up session. Patients were followed up monthly in the first 6 months and subsequently every 3 months for 2 years, or until death or withdrawal from the study.

### End points

The primary endpoint was safety. Safety mainly referred to the severity, frequency and duration of adverse events. Cytokine release syndrome (CRS) was diagnosed and rated against Lee’s criteria [42], and other toxicities were graded per the Common Terminology Criteria of Adverse Events version 5.0. The key secondary endpoint was overall response rate (ORR). ORR was defined as the percentage of patients achieving a stringent complete response (sCR), CR, very good partial response (VGPR) or PR per the IMWG criteria at any time after infusion [43]. Other secondary endpoints included progression-free survival (PFS), duration of response (DOR) and OS. PFS was defined as the period from first infusion to death from any cause or progression, and progression is defined per the IMWG criteria [43]. DOR was defined as the duration from the time when patients achieved a first PR or better to relapse or progression. OS was defined as the time from infusion to death. The exploratory end points were in vivo expansion and persistence of BM38 CAR-Ts.

### Assessment of myeloma cells in bone marrow, including evaluation of BCMA and CD38 expression

Bone marrow samples were divided into 2 tubes. The first tube was used to measure the MM burden in bone marrow. Cells were stained with anti-CD45-V500 (662912; BD), anti-CD19-PE-Cy7 (560728; BD), anti-CD138-APC (347193; BD), anti-CD38-V450 (562444; BD) and anti-CD56-APC-Cy7 (362512; BioLegend) for 30 min. After fixation and permeabilization, the cells were then stained with anti-clambda-PE (555797; BD) and anti-ckappa-FITC (555791; BD). Data were collected using FACS Canto II flow cytometer (BD, USA), and a minimum of 1 × 10^6^ cells were acquired per sample. MM cells were analyzed according to the report by the European Myeloma Network [44]. Minimal residual disease (MRD) was defined according to international consensus guidelines and MRD negativity was defined as less than 0.01% nucleated cells [45].

The second tube was used to detect BCMA and CD38 expression on MM cells. Cells were stained with anti-CD45-V500 (662912; BD), anti-CD19-PE-Cy7 (560728; BD), anti-CD138-V450 (562935; BD), anti-CD56-PerCP-Cy5.5 (560842; BD), anti-CD38-APC (555462; BD) and anti-BCMA-PE (130-117-544; Miltenyi Biotec). BCMA and CD38 expression was reported as the percentage of positive cells in CD45^+^ CD138^+^ CD19^−^ cells. Data were collected using FACSCanto II flow cytometer (BD, USA), and data were analyzed using FlowJo software (TreeStar, USA).

### IHC

MM biopsies were obtained from extramedullary plasmacytoma or bone marrow as formalin-fixed, paraffin-embedded sections. Sections were deparaffinized in xylene, rehydrated in graded alcohol, steamed in an antigen retrieval solution (S1699; Dako, USA) for 30 min, soaked in 30% H_2_O_2_ for 15 min and then blocked with 5% bovine serum albumin for 30 min. Then, the sections were incubated with 1 mg/mL rat-anti-human anti-BCMA antibody (ab17323; Abcam) at room temperature for 60 min followed by HRP-conjugated goat-anti-rat IgG (ab205720; Abcam) at room temperature for 30 min for BCMA staining; or with 1 mg/mL rabbit-anti-human anti-CD38 antibody (ab108403; Abcam) at room temperature for 60 min followed by HRP-conjugated goat-anti-rabbit IgG (ab205718; Abcam) for CD38 staining. The sections were washed three times with PBS and then developed with the DAB detection kit (DAB-0031; MXB, China) according to the manufacturer’s instructions. Nuclei were counterstained with hematoxylin, and images were captured using an Olympus BH-2 microscope (Olympus, Japan).

### Other clinical evaluation

Electrophoresis, immunofixation electrophoresis and 24-h urinary protein analysis were performed by Sebia hydrasys (Sebia, France). Serum and urine free light chain levels were measured using Binding Site. Serum cytokine levels were measured using commercial kits (CellGene, China) according to the manufacturer’s instructions, as previously described [46]. Cytogenetic and genomic aberrations were identified by karyotyping and fluorescence in situ hybridization. ^18^F-FDG positron emission tomography-computed tomography (CT) was used to identify lesions at the time of initial evaluation, and biopsy and IHC were further performed to confirm tumor infiltration plus CD38 and BCMA expression. CT was used to evaluate EMD during follow-up.

### Quantitative PCR

Genomic DNA was isolated from fresh PBMCs obtained at serial time points before and after infusion of BM38 CAR-Ts using the TIANamp Genomic DNA Kit DP340 (Tiangen, China). The primers for BM38 CAR were as follows: the forward sequence was AGAGGAAGATGGCTGTAG; the reverse sequence was CTGCTGAACTTCACTCTC; the probe was FAM-CACATCCTCCTTCTTCTTCTTCTGG-TAMRA (GenScript, China). Vector plasmids with a known CAR copy number were diluted to generate a concentration gradient and used to create a standard curve. All samples were measured three times using Roche LightCycler 96 PCR (Roche, USA). The results are reported as copies per microgram of genomic DNA, with a detection limit of 1000 copies per microgram of genomic DNA.

### Statistical analysis

The statistical test used in the preclinical study is described in the corresponding figure legends. All 23 patients who received infusions were included in the analysis. Descriptive statistics included means with 95% confidence intervals (CIs) or medians with ranges and counts with percentages for categorical variables. Missing data were not imputed. Exact methods (Clopper–Pearson 95% CI) and Fisher’s exact test were used for categorical variables. Continuous variables were compared using unpaired t test or one-way ANOVA. DOR, PFS and OS of patients, and in vivo survival of BM38 CAR-Ts were determined by using the Kaplan–Meier method and compared by utilizing the log-rank test. All analyses were performed with SAS (version 9.4) or GraphPad Prism 7. *P* values (two-tailed) less than 0.05 were considered significant.

## Results

### Preclinical results of BM38 CAR-Ts

We constructed the bispecific BM38 CAR and 38BM CAR by connecting an anti-BCMA scFv and an affinity-optimized anti-CD38 scFv in 4-1BB-containing second-generation formats (Fig. 1a). BM38, 38BM, BCMA and CD38 CARs were stably expressed on lentivirus-transduced T cells from healthy donors, and the final CAR-Ts maintained CD38^+^ expression (Fig. 1b). BM38 CAR-Ts composed 58.5% of CD8^+^ cell population and 39.5% of CD4^+^ cell population, and appeared to be naïve T cells (51.5%) and central memory T cells (26.1%), consistent with the phenotypes of single-target and 38BM CAR-Ts (Fig. 1c; Additional file 1: Fig. S1 for representative staining). BM38, 38BM and CD38-directed CAR-Ts exhibited efficient in vitro expansion equivalent to those of BCMA-directed CAR-Ts and NT cells (Fig. 1d), without observable fratricide.

To explore the anti-MM activities of these CAR-Ts, three MM cell lines, MM.1 s, U266 and RPMI-8226, with heterogeneous BCMA and CD38 expression were used (Additional file 1: Fig. S2). Raji and K562-BCMA cells served as single-positive controls, and K562 cells served as dual-negative controls. Compared to single-target and 38BM CAR-Ts, BM38 CAR-Ts exhibited stronger cytotoxicity and released more interferon γ when coincubated with BCMA^+^CD38^+^ MM cells (Fig. 1e–f). BM38 CAR-Ts also showed comparable activity against BCMA^+^CD38^−^ or BCMA^−^CD38^+^ target cells compared to single-target CAR-Ts. However, BM38 CAR-Ts did not respond obviously to BCMA^−^CD38^−^ K562 cells. In MM xenograft mouse models, BM38 CAR-Ts effectively eradicated MM cells (Fig. 1g) and signified a favorable prognosis than NT cells, BCMA-targeted CAR-Ts or 38BM CAR-Ts (Fig. 1h). All the results demonstrated the specificity and activity of BM38 CAR-Ts against BCMA^+^ or CD38^+^ tumor cells.

### Patients characteristics

As of December 24, 2019, 29 patients with RRMM were screened for eligibility, and 26 patients were initially enrolled. Three patients discontinued the treatment due to rapid disease progression before infusion (*n* = 2) and cell preparation failure (*n* = 1). Twenty-three patients finally received infusions of BM38 CAR-Ts, and a consort diagram is provided in Additional file 1: Fig. S3. The patients treated in the trial received a median of 4 (range 2–9) prior lines of therapy (Table 1, Additional file 1: Table S1). Sixty-one percent of the patients were refractory to their last treatment regimen, and 3 patients received prior autologous stem cell transplantation. Because of osteolytic damage, 52% of the patients scored 2–4 for ECOG performance status. Seventy-four percent had stage II to III disease, 74% had high-risk cytogenetic profiles, and 39% had EMD.

**Table 1:** Characteristics of the patients

All evaluable patients, * n* = 23	Median (range) or No. (%)
Age, year	59 (49–72)
*Gender*	
Male	11 (48)
Female	12 (52)
*ECOG performance status*	
0–2	19 (83)
3–4	4 (17)
*Monoclonal type*	
IgA	6 (26)
IgG	7 (30)
k light chain	3 (13)
λ light chain	4 (18)
others(IgD, IgM)	3 (13)
Extramedullary plasmacytomas	9 (39)
Complex karyotype	5 (22)
Cytogenetic profiles*	17 (74)
*R-ISS stage*	
I	6 (26)
II	6 (26)
III	11 (48)
Time since diagnosis, year	2.9 (0.4–13.4)
*MM burden at enrollment*	
Serum M protein (g/L)	15.9 (0.5–58.9)
Myeloma cells in bone marrow by FCM (%)	11.9 (0.2–56.1)
*Refractory/Relapse at enrollment*	
Refractory to the last treatment	14 (61)
Relapse after the last treatment	9 (39)
Previous therapies	4 (2–9)
Prior ASCT	3 (13)
*Prior proteasome inhibitors*	
Bortezomib	23 (100)
Ixazomib	4 (17)
*Prior immunomodulatory drugs*	
Lenalidomide	13 (57)
Thalidomide	15 (65)
Pomalidomide	1 (4)

ECOG, Eastern Cooperative Oncology Group; FCM, flow cytometry; ASCT, autologous stem cell transplantation

*Includes amplification 1q21, deletion 17p, deletion 13q, *t*(4;14), *t*(11; 14) and *t*(14; 16)

### Safety

CRS occurred in 87% of the patients and was mostly grade 1–2 (65%) per Lee criteria (Table 2) [42]. Four patients received tocilizumab, and three were given glucocorticoids. The median time of CRS onset was 9 days (range 3–19), and the median duration was 7 days (range 1–21). Despite the subgroup analysis limited by the small sample size, CRS severity was associated with MM cells in bone marrow, and peak levels of serum IL-6 and ferritin; CAR-T levels in peripheral blood and infused dose of BM38 CAR-Ts were independent of CRS grades (Additional file 1: Table S2). Four patients had downgraded CRS per the ASTCT scale (Table 2) [47]. Neurotoxicity was not observed.

**Table 2:** Adverse events within 2 months after infusion

Adverse event	Any Grade	Grade 3	Grade 4
*Number of patients (percent)*			
CRS per Lee Criteria	20 (87)	2 (9)	3 (13)
CRS per ASTCT Consensus*	20 (87)	4 (17)	0
CRES	0	0	0
*Hematologic*			
Leukopenia	20 (87)	6 (26)	13 (57)
Neutropenia	22 (96)	3 (13)	17 (74)
Anemia	10 (43)	3 (13)	0
Thrombocytopenia	14 (61)	4 (17)	7 (30)
*Pulmonary*			
Pleural effusion	3 (13)	1 (4)	0
Pulmonary edema	1 (4)	1 (4)	0
*Gastrointestinal*			
Diarrhea	3 (13)	0	0
Vomiting	1 (4)	0	0
*Hepatic*			
AST increase	8 (35)	3 (13)	0
ALT increase	10 (43)	4 (17)	1 (4)
*Nephritic*			
Creatinine increase	3 (13)	0	1 (4)
*Infection*			
Oral candidiasis	1(4)	0	0
Upper respiratory tract infection	2 (9)	0	0
Lung infection	1 (4)	1 (4)	0
Digestive infection	1 (4)	1 (4)	0
*Others*			
Fever	19 (83)	2 (9)	0
Hypotension	1 (4)	0	0
Hypoxemia	3 (13)	1 (4)	1 (4)
Sinus tachycardia	8 (35)	1 (4)	0
Hypocalcemia	15 (65)	2 (9)	1 (4)
Hypokalemia	6 (26)	0	0
Hyponatremia	5 (22)	0	0

CRS, cytokine release syndrome; CRES, CAR T-cell-related encephalopathy syndrome; ALT, alanine aminotransferase; AST, aspartate aminotransferase

*CRS was downgraded per ASTCT consensus for the following reasons: patient 5 for grade 3 hepatotoxicity, patient 7 for grade 4 coagulopathy, patient 9 for grade 4 nephrotoxicity and patient 19 for emergency thoracentesis due to fatal pleural effusion

Hematologic toxicities were the most common events, including neutropenia in 96% of the patients, leukopenia in 87%, anemia in 43% and thrombocytopenia in 61% (Table 2). Given the expected toxicities of lymphodepleting chemotherapy, grade 3–4 leukopenia was observed in 20 patients, and 60% recovered to an absolute leukocyte count of at least 2000 cells per cubic millimeter (≤ grade 2) by month 1 (Additional file 1: Fig. S4). Grade 3–4 neutropenia occurred in 19 patients after infusion, and 60% recovered to an absolute neutrophil count of at least 1000 cells per cubic millimeter (≤ grade 2) within 1 month. Eleven patients suffered grade 3–4 thrombocytopenia, and 45% recovered to a platelet count of at least 50 000 per cubic millimeter (≤ grade 2) by month 1. Grade 3 or higher anemia was infrequent, and all 3 patients recovered to the hemoglobin concentration of at least 80 g per liter by month 3. Delayed recovery from severe cytopenia was observed. The median time from infusion to recovery from ≤ grade 2 leukopenia, neutropenia or thrombocytopenia was 28 days (range 14–90), 28 days (range 13–90) and 44 days (range 14–150), respectively. Hepatotoxicity, manifested as elevated alanine aminotransferase and aspartate aminotransferase levels, was noted in 10 patients (43%), and all cases resolved by month 1. Three patients were observed with nephrotoxicity manifested by an elevated serum creatinine level, and one required hemodialysis.

Hypoimmunoglobulinemia existed in 83% of the patients before CAR-T cell therapy, became more serious after infusion and lasted during follow-up, except in patient 12 and 14, whose serum immunoglobulin levels recovered to the normal levels in month 5 and month 4 (Additional file 1: Fig. S5). To prevent infections, intravenous immunoglobulin at a dosage of 400 mg/kg every 2–4 weeks was routinely administrated to treated patients for 3 months post-infusion. Five patients experienced infectious diseases by month 2 after infusion, and two needed additional intravenous antibiotics. All the toxicities were manageable.

Two patients died during follow-up. Patient 1 recovered from grade 3 leukopenia and grade 2 neutropenia by month 1, and achieved a sCR in month 4. The patient had hypogammaglobulinemia prior to CAR-T cell therapy, but exhibited recovery to normal levels of immunoglobulins A and M in month 6. The patient suffered from pulmonary infections, which eventually developed into septic shock, and died on day 459. Patient 7 was a 66-year-old female with hypertensive diseases for 10 years. She attained a VGPR in month 2, and recovered from grade 4 leukopenia, neutropenia and thrombocytopenia by month 3. The patient developed an abrupt and severe headache, vomiting and disturbance of consciousness on day 132 and was diagnosed with acute intracranial hemorrhage; stage III hypertensive disease. The patient eventually died after emergency treatment; the outcome was considered unrelated to the study treatment.

### Efficacy

**Fig. 2:**
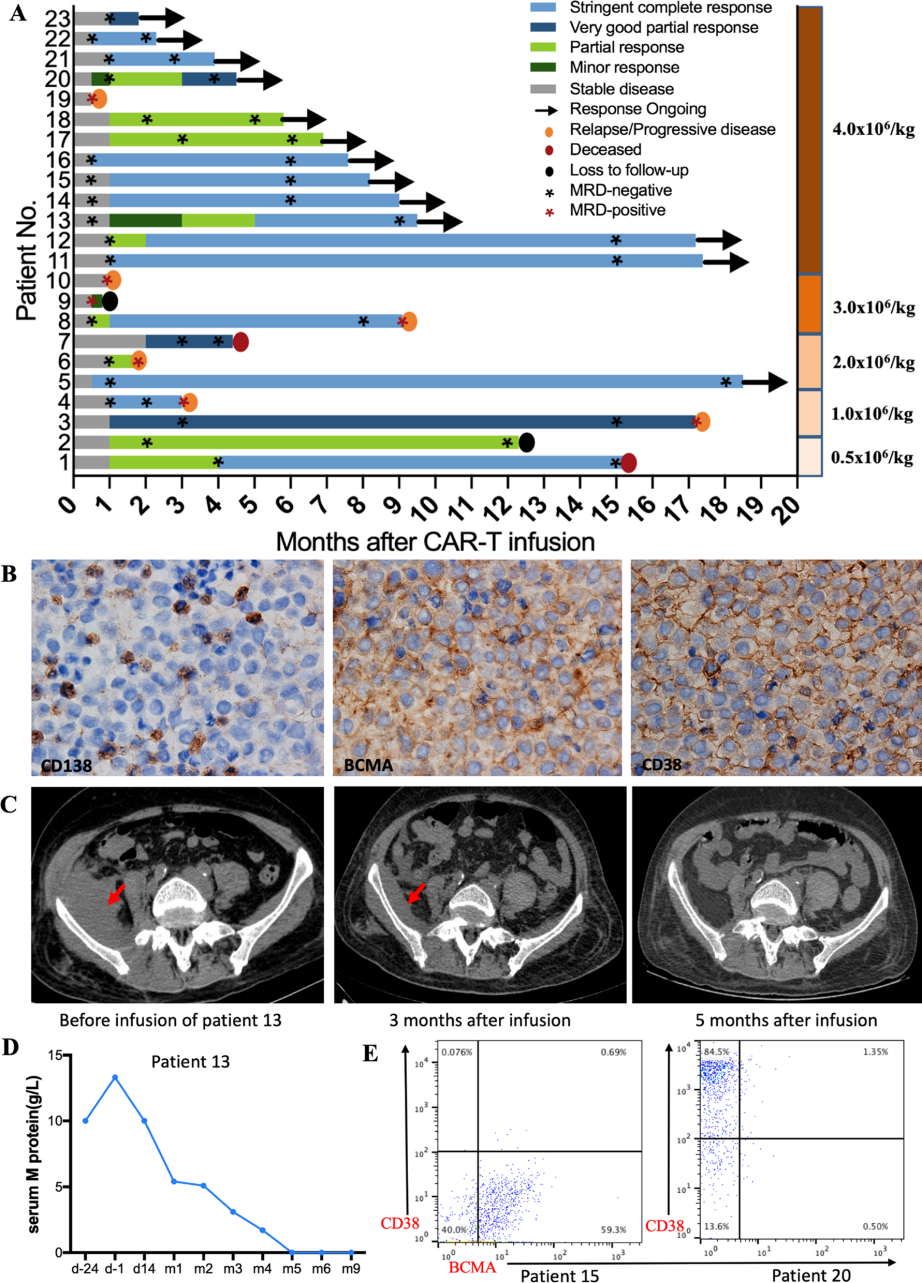
Clinical responses mediated of BM38 CAR-Ts. **a** Swimmer plot for the 23 patients treated in the study. Two patients were enrolled in the two low-dose groups for the consideration of risk–benefit trade-off. **b** Immunohistochemical staining of patient 13’s right parailiac mass before treatment, showing involvement of MM cells and positive expression of BCMA and CD38. **c** Computed tomography scans of patient 13 showing a large right parailiac plasmacytoma at enrollment that was partially eliminated in month 3 and completely disappeared in month 5. **d** Changes of serum M protein in patient 13 during BM38 CAR-T treatment. **e** BCMA and CD38 expression on MM cells in patient 15 and 20 at baseline. Representative staining and gating of MM cells are shown in Additional file 1: Fig. S6

Eighty-seven percent of the 23 patients responded, including 12 (52%) achieving a sCR, 4 (17%) achieving a VGPR and 4 (17%) achieving a PR (Fig. 2a and Additional file 1: Table S3). The median time to the first PR or better was 1.0 month (range 0.5 to 2.0), and responses improved over time in some patients.

Patient 13 had 2.98% MM cells in bone marrow at baseline, among which 80.7% expressed BCMA and 100% expressed CD38 (Additional file 1: Fig. S6). The patient had a right parailiac mass at enrollment, biopsy showed MM involvement, and IHC confirmed positive expression of BCMA and CD38 (Fig. 2b). After BM38 CAR-T infusion, MM cells in bone marrow were undetectable on day 14. EMD was eliminated gradually, as indicated by CT imaging (Fig. 2c). The serum M protein level of the patient decreased precipitously and became undetectable in month 5 (Fig. 2d). Patient 13 attained a PR in month 3 and had a deepening response to sCR in month 5.

All 20 responders (100%) achieved MRD-negativity (≤ 10^–4^ nucleated cells), as determined by flow cytometry. CD38 expression was absent on MM cells from patient 15 at baseline, BCMA expression was lacking on MM cells from patient 20 (Fig. 2e). Both the patients attained MRD-negativity after infusion, indicating that BM38 CAR-Ts could effectively eradicate MM cells with heterogeneous BCMA and CD38 expression in vivo. Although subgroup analyses were limited by small sample sizes, clinical responses were not significantly influenced by baseline tumor BCMA and CD38 levels, MM cells in bone marrow, serum M protein or dose of infused CAR-Ts (Additional file 1: Fig. S7 and Table S3).

EMD was eliminated completely in 56% and partially in 33% of 9 patients as confirmed by CT (Additional file 1: Fig. S8). However, EMD did not show a significant effect on the patients’ outcomes (Additional file 1: Fig. S9).

**Fig. 3:**
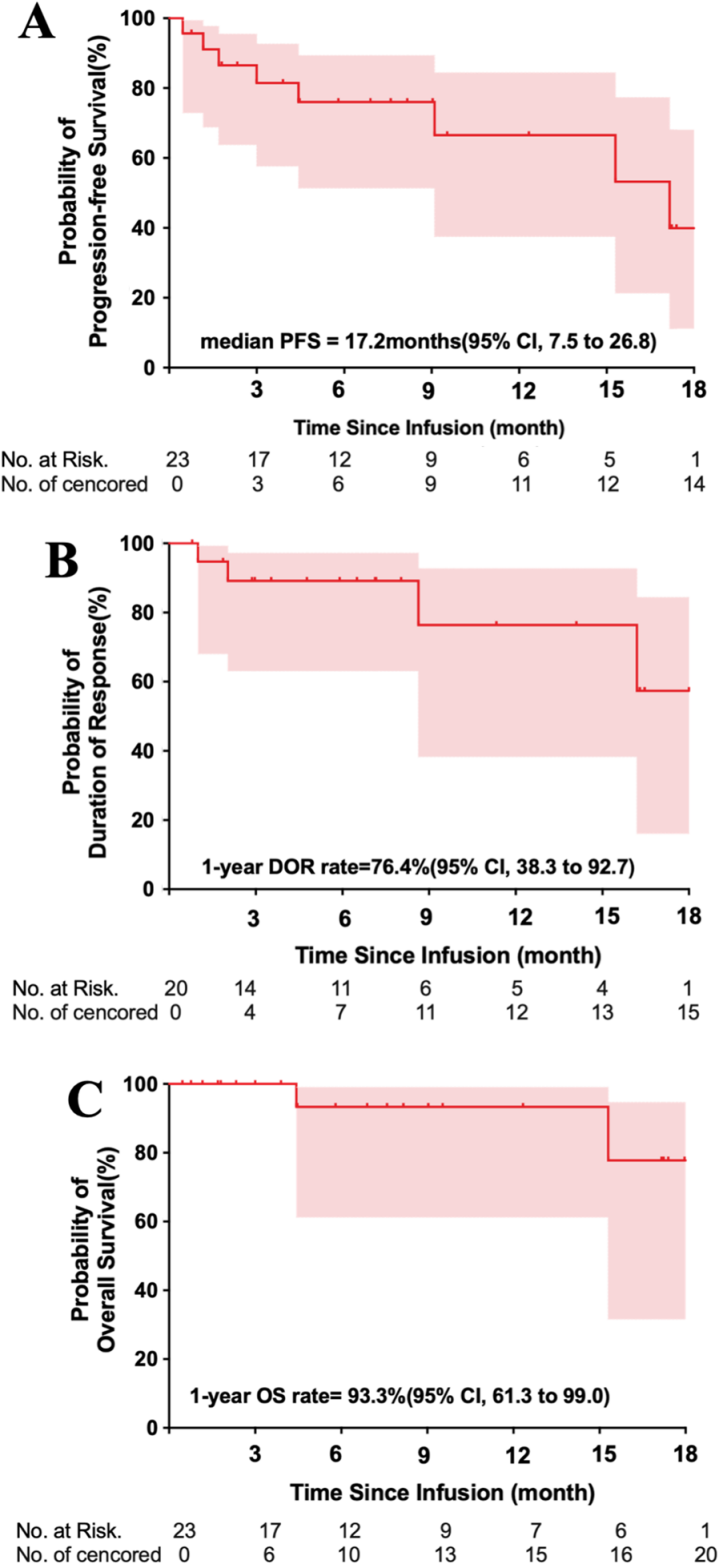
Progression-free survival, duration of response and overall survival. Kaplan–Meier curves for PFS (**a**) and OS (**c**) of the 23 treated patients, and DOR (**b**) of the 20 patients who responded. The shaded region indicates the 95% CI

At a median follow-up of 9.0 months (range 0.5 to 18.5), 13 patients had ongoing responses, and 92% were treated with the dose of 4.0 × 10^6^ CAR-Ts/kg. Two responders relapsed from a sCR when MM cells in bone marrow still maintained BCMA and CD38 expression. The overall median PFS was 17.2 months (95% CI 7.5 to 26.8) (Fig. 3a). The median DOR and OS were not reached, and the 1-year DOR and OS rate were 76% (95% CI 38% to 93%) and 93% (95% CI 61% to 99%), respectively (Fig. 3b, c). The median PFS was 9.1 months at the dose of 0.5 × 10^6^ to 3.0 × 10^6^ BM38 CAR-Ts/kg, and not reached at the dose of 4.0 × 10^6^ CAR-Ts/kg (Additional file 1: Table S3).

### Expansion and persistence

**Fig. 4:**
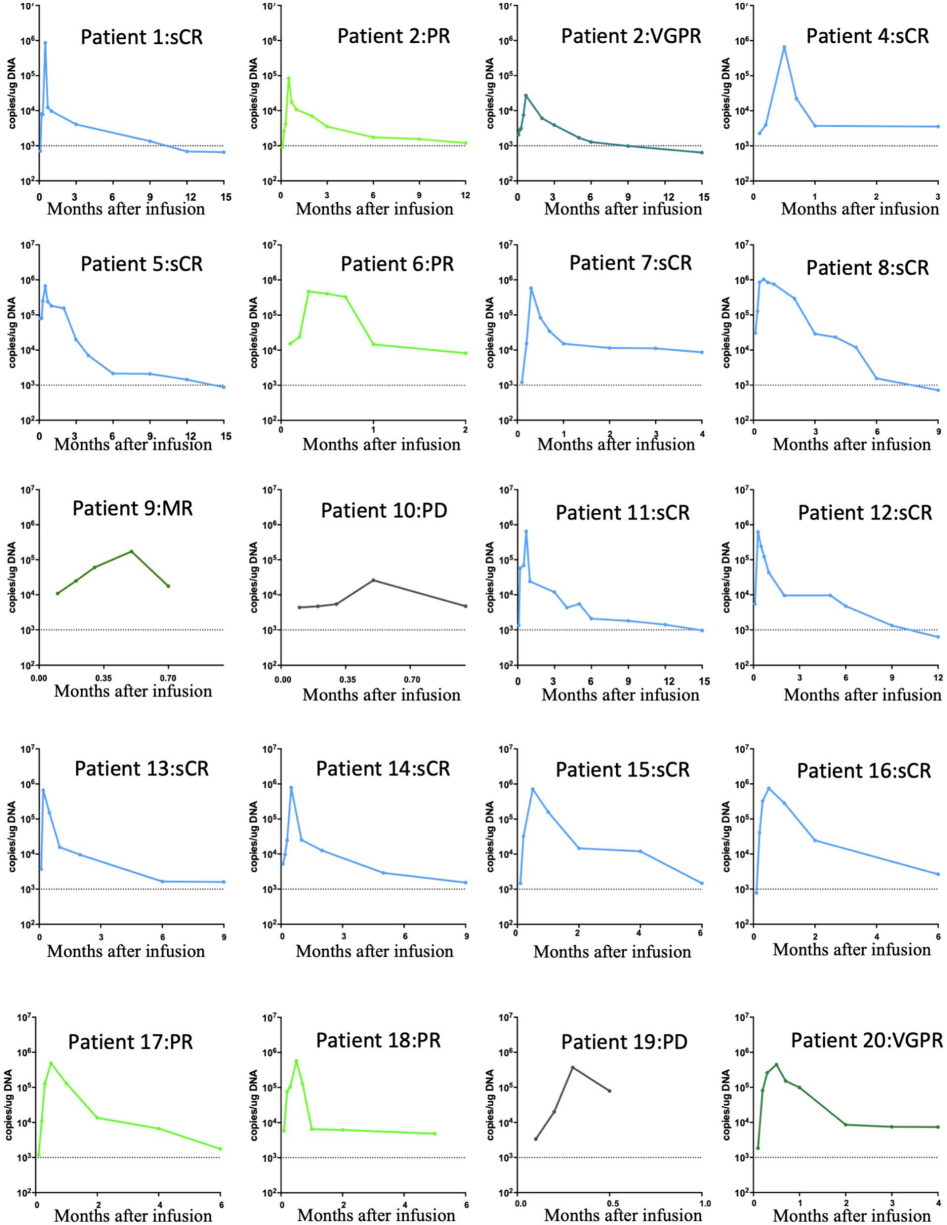
In vivo expansion of BM38 CAR-Ts by qPCR in the first 15 months. BM38 CAR-T expansion was measured as copies/µg of genomic DNA by qPCR in peripheral blood in 20 evaluable patients. The detectable threshold was 1000 copies/µg of genomic DNA

BM38 CAR + T cells accounted for 29.8% (range 12.0% to 60.0%) of the final infused cell products, and the median expansion folds were 32.4 (range 11.4 to 72.9) on day 5 after lentiviral transduction (Additional file 1: Table S4). BM38 CAR-Ts in peripheral blood peaked at a median of 11 days (range 4 to 21 days) after infusion (Fig. 4). In vivo expansion of BM38 CAR-Ts was measured as CAR copies per microgram of genomic DNA by qPCR, andin vivo exposures were measured as area under the curve of CAR copies per microgram of genomic DNA in the first 28 days after infusion (AUC0-28 d﻿ays).

**Fig. 5:**
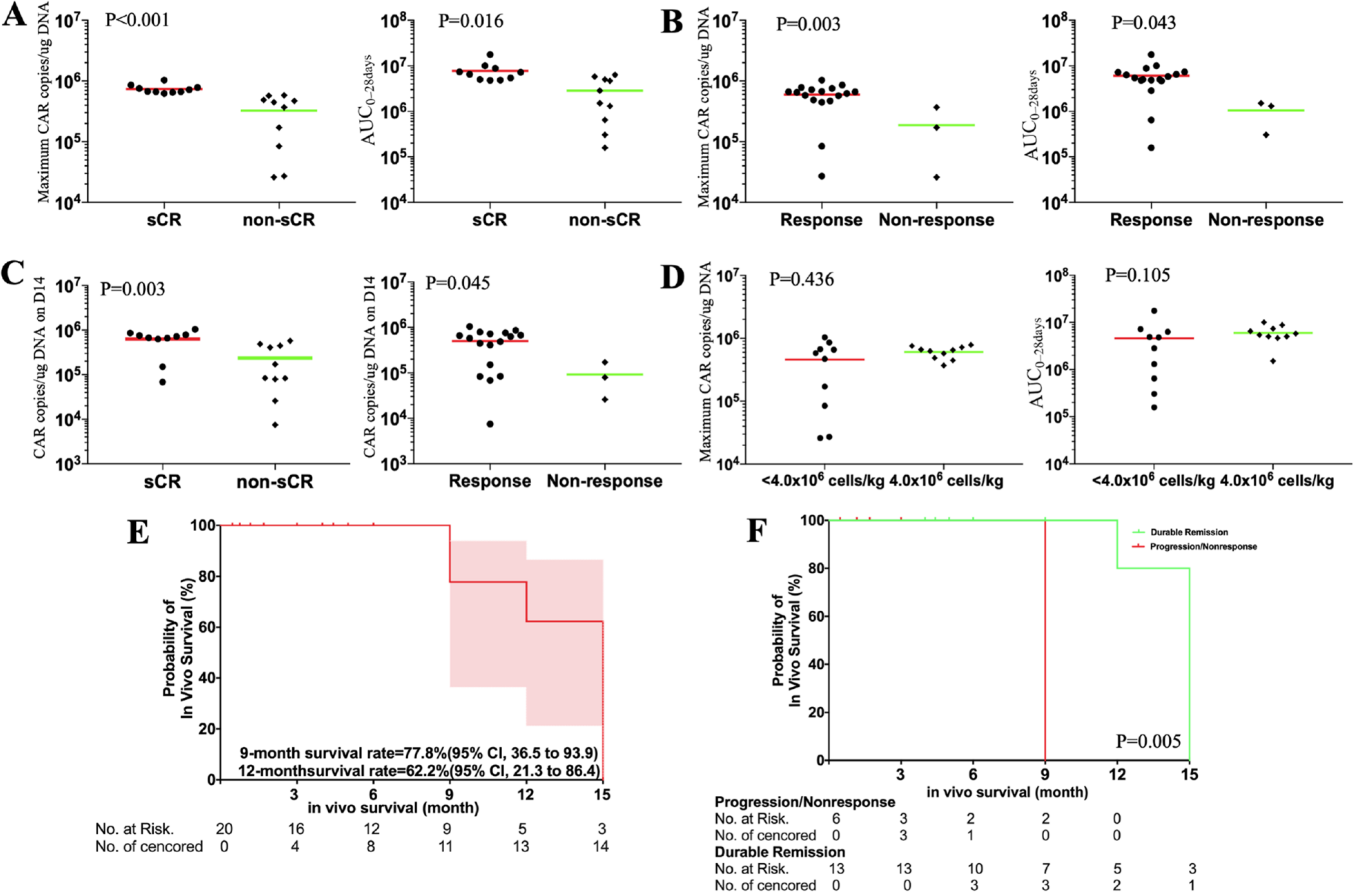
Expansion and persistence of BM38 CAR-Ts associated with clinical response. The peak expansion and AUC_0-28 days_ of BM38 CAR-Ts were associated with a sCR (**a**) and clinical response (**b**), but not with infused dose (**d**). Patients who achieved a sCR and responded had higher peak expansion on day 14 (**c**). An unpaired t test was used. Kaplan–Meier survival curves of in vivo BM38 CAR-Ts, overall (**e**) and by responses (**f**). The log-rank test was used

Patients who achieved a sCR had higher peak expansion and in vivo exposures (Fig. 5a), which was also observed in responders (Fig. 5b). Among responders, in vivo exposures did not show statistical significance, whereas superior peak expansion was observed in the patients who achieved a sCR (Additional file 1: Fig. S10a, b). Day 14 was a vital point of time to monitor in vivo concentration of BM38 CAR-Ts because it was related to clinical responses (Fig. 5c). Peak expansion and in vivo exposures of BM38 CAR-Ts were independent of the infused dose (Fig. 5d), possibly because it was incremental at a slow and small step. The MM burdens did not show an association with the in vivo expansion of BM38 CAR-Ts (Additional file 1: Fig. S10c–f). Persistence was durable, with 77.8% (95% CI 36.5% to 93.9%) and 62.2% (95% CI 21.3% to 86.4%) of the 20 evaluable patients having detectable BM38 CAR copies at 9 and 12 months, respectively (Fig. 5e). Longer in vivo persistence of BM38 CAR-Ts was observed in the patients who had ongoing responses (Fig. 5f).

### Computational docking of BM38 CAR to target molecules

**Fig. 6:**
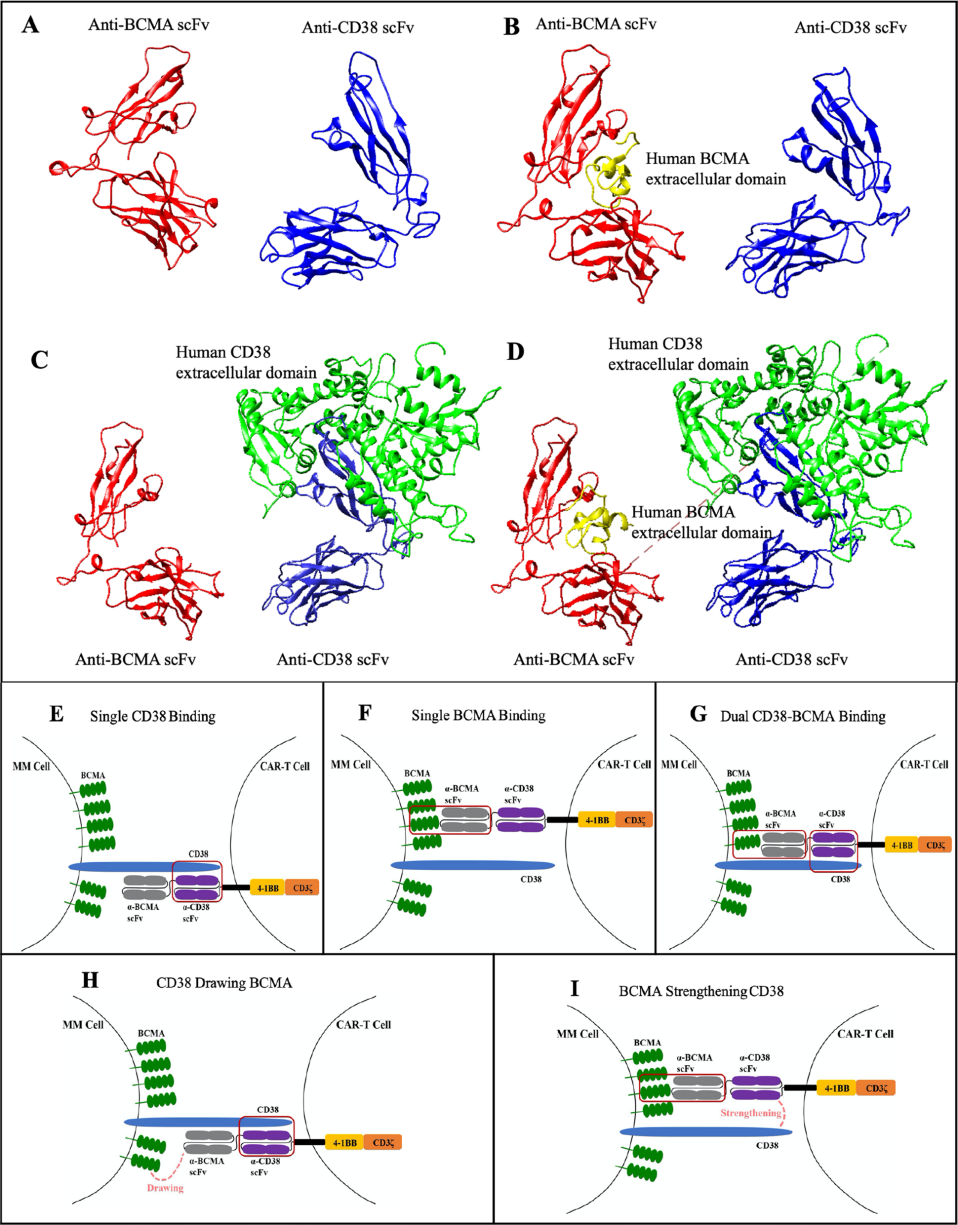
Predictive docking patterns of BM38 CAR to MM cells. **a** Hypothetical structure of the anti-BCMA scFv and anti-CD38 scFv joined with an (EAAAK)_3_ linker (not shown). **b** Most favorable docking models of the anti-BCMA scFv with 51 amino acid residues of the extracellular domain of BCMA (2kn1.pdb); **c** Most favorable docking models of the anti-CD38 scFv with 257 amino acid residues of the extracellular domain of CD38 (1yh3.pdb); **d** Dual docking of BM38 CAR with BCMA and CD38. **e** Theoretical single combination pattern of the anti-CD38 scFv and CD38 on MM cells. **f** Theoretical single combination pattern of the anti-BCMA scFv and BCMA on MM cells. **g** Theoretical double-binding pattern of the bispecific BM38 CAR to BCMA and CD38 on MM cells. **h–i** The CD38 extracellular domain contains 257 amino acids and the BCMA extracellular chain consists of only 54 amino acids. The anti-BCMA scFv and anti-CD38 scFv contain 246 and 249 amino acids, respectively. Theoretically, CD38 binding might assist the anti-BCMA scFv in binding to BCMA on myeloma cells, and BCMA coalescence would reversely strengthen CD38 binding

To investigate how BM38 CAR theoretically interacts with target molecules on MM cells, we created a BM38 CAR structural model (Fig. 6a) using the protein-structure modeling *SWISS-MODEL Server* [48]. We also used computational modeling tools to predict the docking patterns of BM38 CAR with target molecules [49]. BM38 CAR recognized BCMA and CD38 individually (Fig. 6b, c), and facilitated synergistic activation and functionality when both BCMA and CD38 were encountered simultaneously (Fig. 6d). In addition to the three classic binding patterns (Fig. 6e–g), CD38 binding might assist the anti-BCMA scFv in connection with BCMA on myeloma cells (Fig. 6h), and BCMA coalescence could reversely strengthen CD38 binding (Fig. 6i) in view of their extracellular domains. The synergistic binding might contribute to the enhanced cytotoxicity of BM38 CAR-Ts to BCMA^+^ CD38^+^tumor cells.

## Discussion

MM is a phenotypically heterogeneous malignancy, with multifarious subclones in individual patients, which makes it susceptible to escape from single-target immunotherapy [50–52]. MM cells are BCMA^−^ in a minority of cases [53], and BCMA downregulation or loss has been observed in relapsed patients after BCMA-directed CAR-T therapy [10, 12, 29, 36], highlighting the rationale for targeting an additional antigen. CD38 is highly and uniformly expressed on MM cells [37, 38], and is also present on some immunosuppressive cell populations [54, 55]. Inspired by these findings, we, to our knowledge, are the first to construct a bispecific CAR targeting BCMA and CD38 (BM38 CAR), as an exploratory therapy for patients with RRMM. BM38 CAR-Ts were well tolerated and displayed significant clinical activity in patients with RRMM who had unfavorable cytogenetic profiles and poor performance status, with an ORR of 87%, a sCR rate of 52%, median PFS of 17.2 months and a 1-year OS rate of 93%. Excellent responses were also seen in patients with EMD.

BCMA-targeted CAR-Ts hold the most promising potential to induce deep and durable remission in patients with RRMM, comparing favorably with current FDA-approved salvage therapies [56, 57] and investigational agents [58]. Daratumumab, the first fully human anti-CD38 monoclonal antibody (mAb), in combination with bortezomib and dexamethasone, resulted in an ORR of 83%, CR rate of 19% and 1-year PFS rate of 61% in patients (*n* = 251) treated with a median of 2 (range 1–9) prior lines of therapy [56]. Isatuximab, a novel chimeric anti-CD38 mAb, in combination with pomalidomide and dexamethasone, attained an ORR of 60%, CR rate of 5% and median PFS of 11.5 months in patients (*n* = 154) treated with a median of 3 (range 2–4) prior regimens [57]. Belantamab mafodotin, the first-in-class BCMA-directed immunoconjugate, showed an ORR of 34% and median PFS of 5.9 months in patients (*n* = 99) treated with a median of 3 (range 1–11) prior regimens [58]. BCMA-directed CAR-Ts, bb2121, achieved an ORR of 73%, CR rate of 33% and median PFS of 8.8 months in more heavily pretreated patients (*n* = 128) treated with a median of 6 (range 3–16) prior regimens [12]. JNJ-4528, biepitope BCMA-targeted CAR-Ts, was reported to produce an ORR of 97%, CR rate of 67% and 1-year PFS of 77% in patients (*n* = 97) treated with a median of 6 prior lines [15]. Although the patients in our study, treated with a median of 4 (range 2–9) prior therapies, were less heavily pretreated than patients in CRB-401 and CARTITUDE-1, BM38 CAR-Ts exhibited an appreciable responsive rate, depth and durability. Our results demonstrated that CAR-Ts could be applied as a more frontline therapy for patients with MM. The patients had not been previously exposed to CD38- or BCMA-targeted immunotherapy, which is one limitation of the study. Further trails will investigate the efficacy of BM38 CAR-Ts in this population.

Eighty-seven percent of the patients experienced CRS, with 22% of grade 3–4 per Lee’s criteria. Regardless of the grading criteria, CRS is one of the most common adverse events (76%-100%) after single BCMA-targeted CAR-T therapy, with the incidence rate of grade 3–4 CRS ranging from 5 to 41% [10–14, 16]. Consistent with previous reports [10, 14], we found that CRS severity was also associated with the myeloma burden in bone marrow, and peak levels of serum IL-6 and ferritin (Additional file 1: Table S1). CRS occurred at a median of 9 days post-BM38 CAR-T infusion, which was coincident with the timing observed for LCAR-B38M CAR-Ts [13, 14] and the sequential infusion of anti-BCMA and anti-CD19 CAR-Ts [18], but later than that observed for bb2121 [11] and CART-BCMA cells [16]. The dose of infused CAR-Ts may be the key determinant of the onset time of CRS. No neurotoxicity was observed, for the potential explanations that BM38 CAR-Ts trafficked into only bone marrow and extramedullary plasmacytoma where MM cells restrictively existed. The observation was also limited by the small sample size and the low dose of infused CAR-Ts.

High-grade hematologic toxicities were common and somewhat persistent in the study. Grade 3–4 leukopenia, neutropenia, thrombocytopenia and anemia were observed in 83%, 87%, 45% and 13% of 23 treated patients, respectively. All the patients recovered to leukopenia, neutropenia and anemia of grade 2 or better by month 3, and to thrombocytopenia of grade 2 or better by month 5. The hematologic toxicities were partially related to lymphodepleting regimens, and were also possibly correlated with the potential effects of the additional affinity-optimized anti-CD38 CAR on HPCs. Additional studies are required to assess the roles of the anti-CD38 scFv in BM38 CAR. Hepatotoxicity was notable in our study, but it varied significantly (12%-100%) in previous studies of BCMA-targeted CAR-Ts [9–16], and was not reported in studies of CD38-directed daratumumab monotherapy [59–61]. The liver dysfunction was unlikely to be an on-target off-tumor effect; it was probably part of a systemic inflammatory syndrome. Overall, BM38 CAR-Ts showed a manageable safety profile.

Patients treated with the high dose of BM38 CAR-Ts attained longer PFS (Additional file 1: Table S2), in line with previous studies [9–12]. At the dose of 4.0 × 10^6^ BM38 CAR-Ts/kg, 92% attained a clinical response, 62% achieved a sCR and all 12 responders maintain ongoing remission at a median follow-up of 218 days. The infused doses of BM38 CAR-Ts did not show an association with CRS severity. Taken together, 4.0 × 10^6^ BM38 CAR-Ts/kg with optimal efficacy and manageable toxicities was a suitable dose in the expansion phase.

The in vivo expansion and survival of BM38 CAR-Ts were positively related to clinical response (Fig. 5). BM38 CAR utilized the fully human anti-BCMA scFv and the humanized anti-CD38 scFv, partially explaining the long-term in vivo persistence. Recipient anti-CAR immune responses could limit the in vivo durability of CAR-Ts [14, 62, 63], but it was not estimated in the study, which was another limitation of the study.

Bispecific or multispecific CARs hold the potential advantages to eradicate heterogeneous tumor cells [64–66]. However, failure to respond and relapse with positive or negative antigens remain challenges. These facts were also observed in our study. Responders, especially those who attained a sCR, showed superior in vivo expansion of BM38 CAR-Ts (Fig. 5a, b). CAR-Ts persisted for a longer time in patients who remained ongoing remission (Fig. 5f). These results suggest that promoting CAR-T expansion and extending CAR-T persistence in vivo is a key strategy for improvement. Moreover, combination with other therapies, such as transplantation [67], BTK inhibitors [68] or anti-PD-1 antibodies [69], may be beneficial to enhance CAR-T functionality and improve the durability of responses. More clinical trials are needed to verify these hypotheses.

## Conclusion

Our study demonstrated for the first time that the humanized bispecific BM38 CAR-Ts targeting BCMA and CD38 were feasible, safe and significantly effective in patients with RRMM. BM38 CAR-Ts showed significant in vivo persistence, deep and durable responses and effective elimination of EMD. The preliminary results require confirmation in future multicenter clinical trials. The additional roles of the CD38-targeted scFv warrant further in-depth investigation.

## Supplementary Information

**Additional file 1**. Supplementary figures and tables.

## Data Availability

All data relevant to the study are included in the article or uploaded as Additional files.

## Abbreviations

BCMA: B-cell maturation antigen
CAR-Ts: Chimeric antigen receptor-T cells
CRS: Cytokine release syndrome
CIs: Confidence intervals
CT: Computed tomography
DOR: Duration of response
ECOG: Eastern Cooperative Oncology Group
EMD: Extramedullary disease
E:T: Effector-to-target
HPC: Hematopoietic progenitor cell
IL: Interleukin
IHC: Immunohistochemistry
IMWG: International Myeloma Working Group
MRD: Minimal residual disease
mAb: Monoclonal antibody
NT: Nontransduced T
OS: Overall survival
ORR: Overall response rate
PBMCs: Peripheral blood mononuclear cells
PFS: Progression-free survival
RR: Refractory or relapsed
scFv: Single-chain variable fragment
sCR: Stringent complete response
VGPR: Very good partial response
