# Supplementary material for: A bispecific CAR-T cell therapy targeting BCMA and CD38 in relapsed or refractory multiple myeloma
Source: J Hematol Oncol. 2021 Oct 9;14:161. doi: 10.1186/s13045-021-01170-7 (PMC8501733; doi:10.1186/s13045-021-01170-7)
Supplement: Supplementary file 1 — Additional file 1. Supplementary figures and tables. [file 13045_2021_1170_MOESM1_ESM.pdf]

Additional file 1

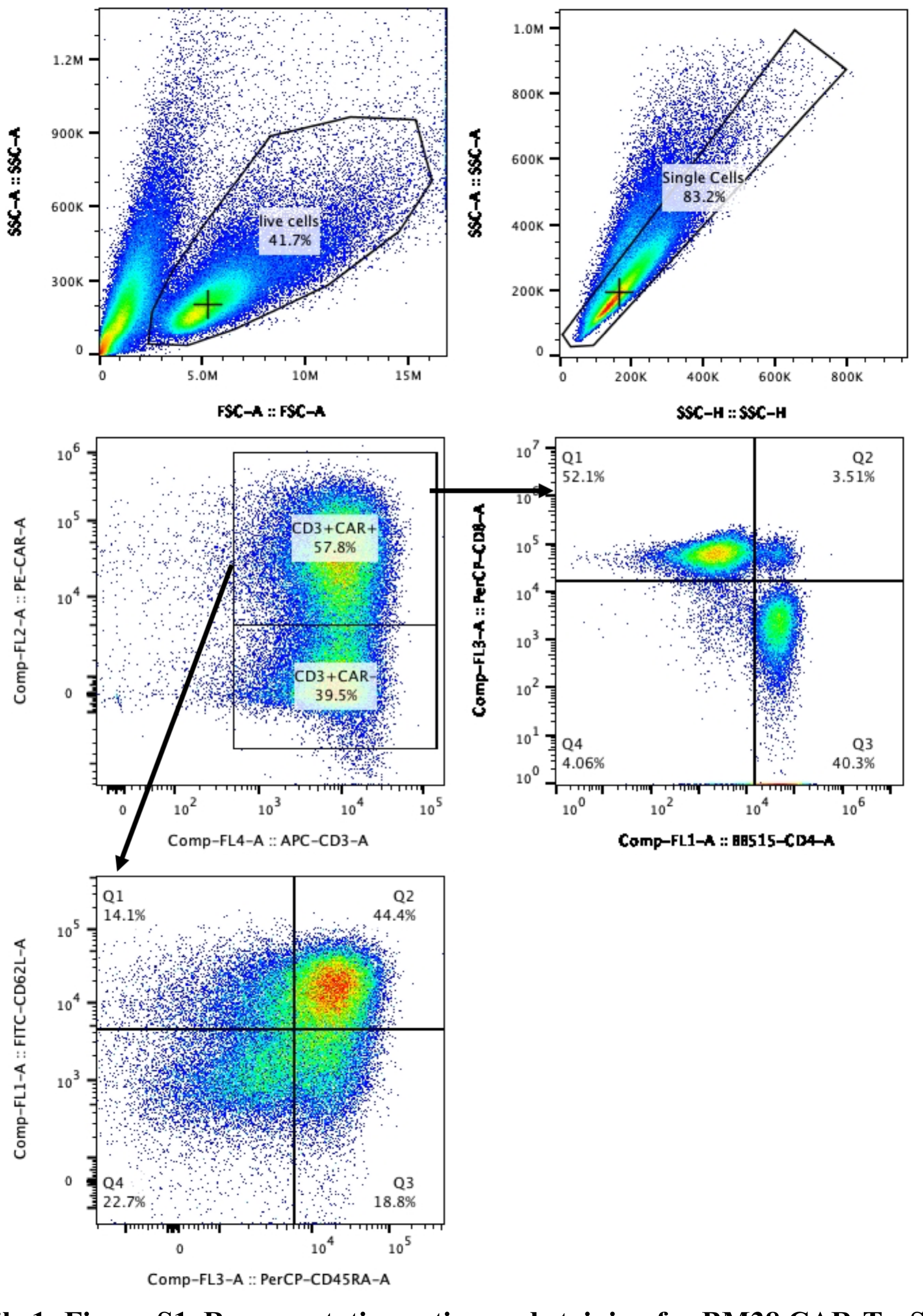

Additional file 1: Figure S1. Representative gating and staining for BM38 CAR-Ts. Staining is shown for BM38 CAR-Ts from a healthy donor, day 10 after lentiviral transduction. Cells are gated by forward and side scatter, then singlets, then CAR^+CD3^+ T cells. The % of $CD3^+$ T cells expressing CAR was calculated as CAR^+CD3^+ cells/ (CAR^+CD3^+ cells + CAR^-CD3^+ cells) (i.e. in this example 57.8% / (57.8% + 39.5%) = 59.4%). CAR-Ts were further divided by CD4 plus CD8, or CD62L plus CD45RA. BM38 CAR was identified using biotinylated human BCMA and streptavidin-PE.

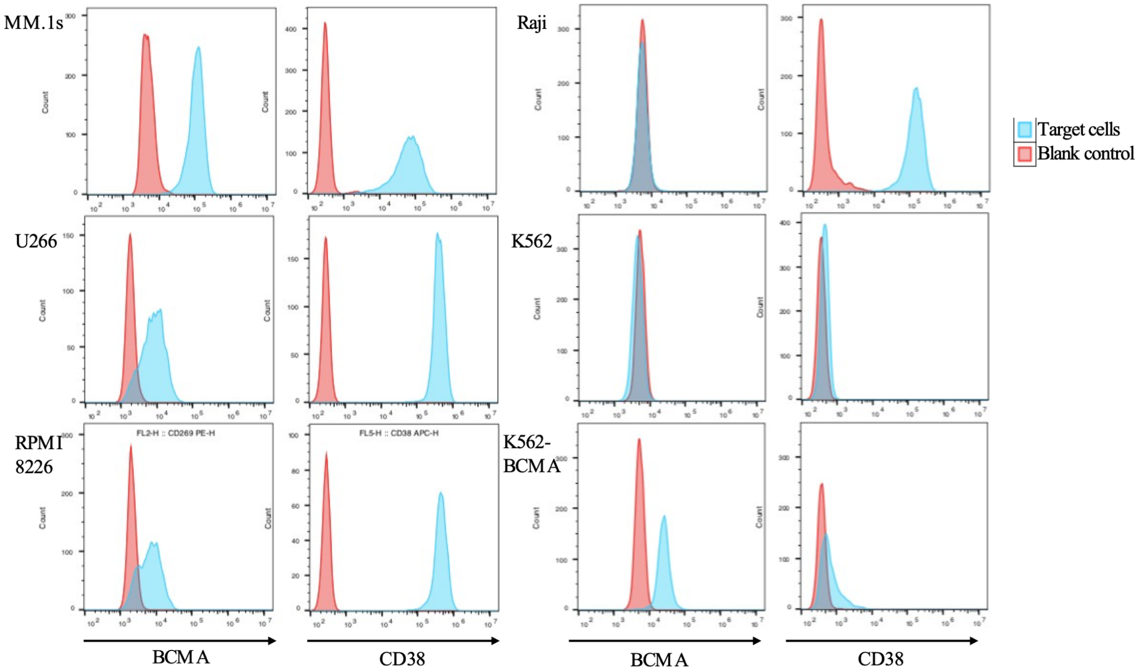

Target cells	MM.1s	U266	RPMI 8226	Raji	K562	K562-BCMA	BM38 CAR-Ts
BCMA MFI	1.15x10^{5}	1.04x10^{4}	8079	5718	4496	2.44x10^{4}	2726
Ratio	22.52	2.04	1.58	1.12	0.88	4.78	0.53
**Qualitative**	**BCMA^{+++}**	**BCMA^{+}**	**BCMA^{+}**	**BCMA^{-}**	**BCMA^{-}**	**BCMA^{++}**	**BCMA^{-}**
CD38 MFI	7.83x10^{4}	4.23x10^{5}	4.52x10^{5}	1.53x10^{5}	475	809	4.89x10^{4}
Ratio	121.96	658.88	693.25	238.32	0.74	1.26	76.17
**Qualitative**	**CD38^{+}**	**CD38^{+++}**	**CD38^{+++}**	**CD38^{++}**	**CD38^{-}**	**CD38^{-}**	**CD38^{+}**

Additional file 1: Figure S2. Expression and qualitative intensity of BCMA and CD38 on target cells. Blank controls were respective target cells without staining using the anti-BCMA or anti-CD38 antibody. BCMA and CD38 qualitative expression was judged by the ratio compared to their negative controls (Raji and K562 for BCMA, mean MFI: 5107; K562 and K562-BCMA for CD38, mean MFI: 642). MFI: mean fluorescence intensity.

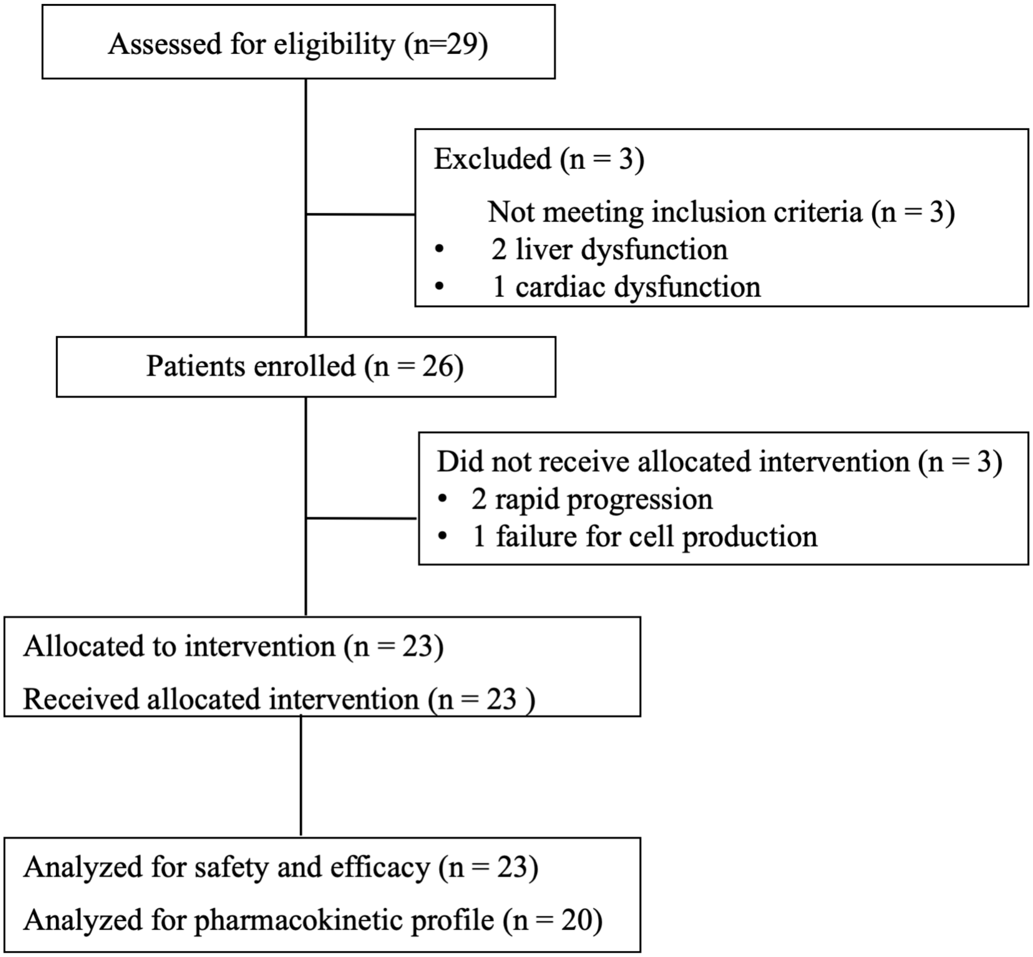

Additional file 1: Figure S3. Consort diagram. Pharmacokinetic profiles weren't available for patient 21, 22 and 23 because of instrument failure for qPCR.

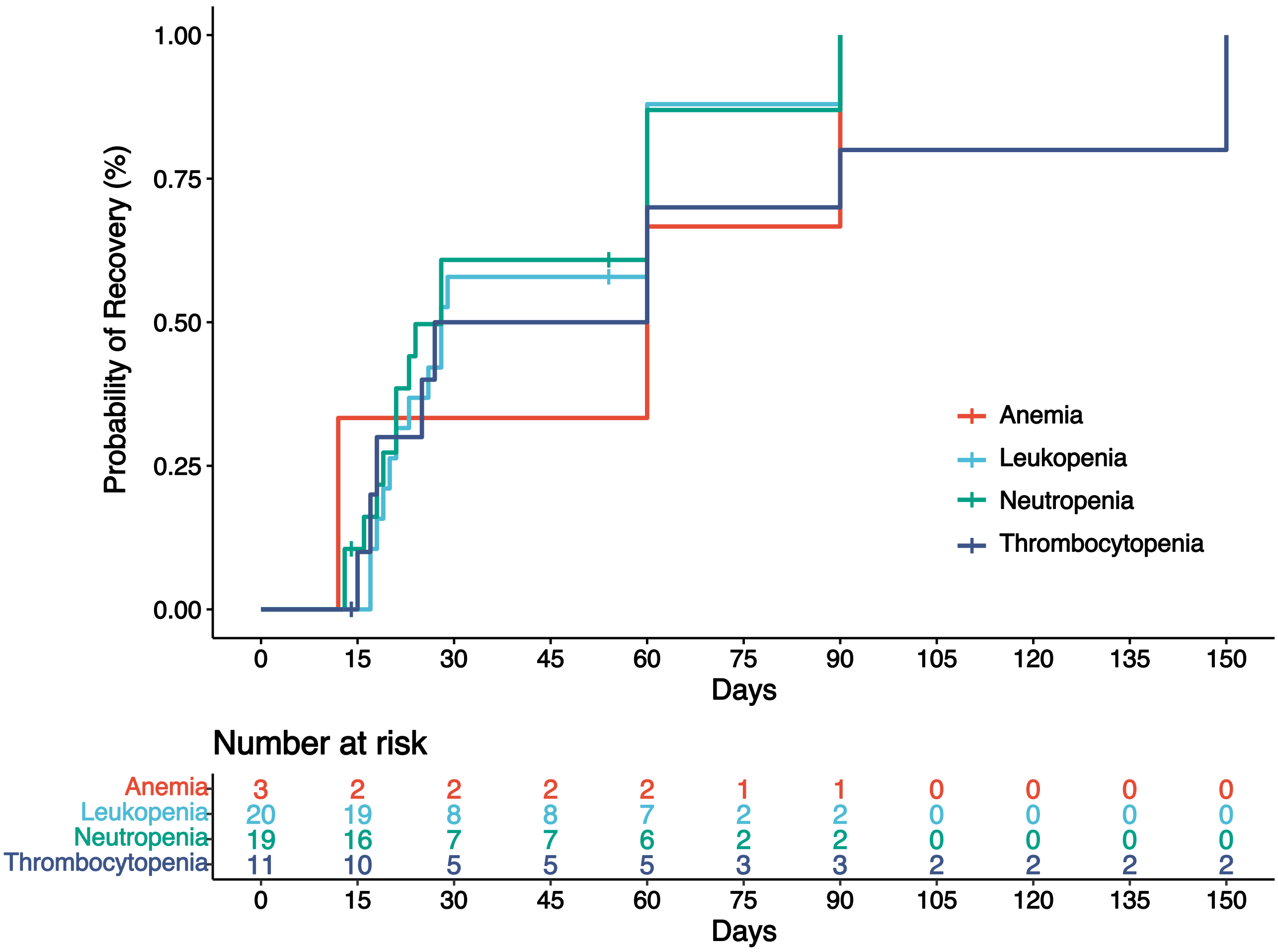

Additional file 1: Figure S4. Time to Recovery of Grade 3/4 Cytopenias. Patients with grade 3/4 cytopenias (absolute leukocyte counts < 2000 cells/µL (n=19), absolute neutrophil counts < 1000 cells/µL (n=20), hemoglobin concentration < 80 grams per liter (n=3), or platelets < 50,000/µL (n=11)) after infusion are included. Recovery is defined as cytopenias of grade 2 or better (absolute leukocyte counts ≥ 2000 cells/µL, absolute neutrophil counts ≥ 1000 cells/µL, hemoglobin concentration ≥80 grams per liter, or platelets ≥ 50,000/µL). Time to recovery is defined as the time from infusion to the first time when recovery criteria was met. Patient 19 and 23 were censored due to progressive disease and limited follow-up, respectively.

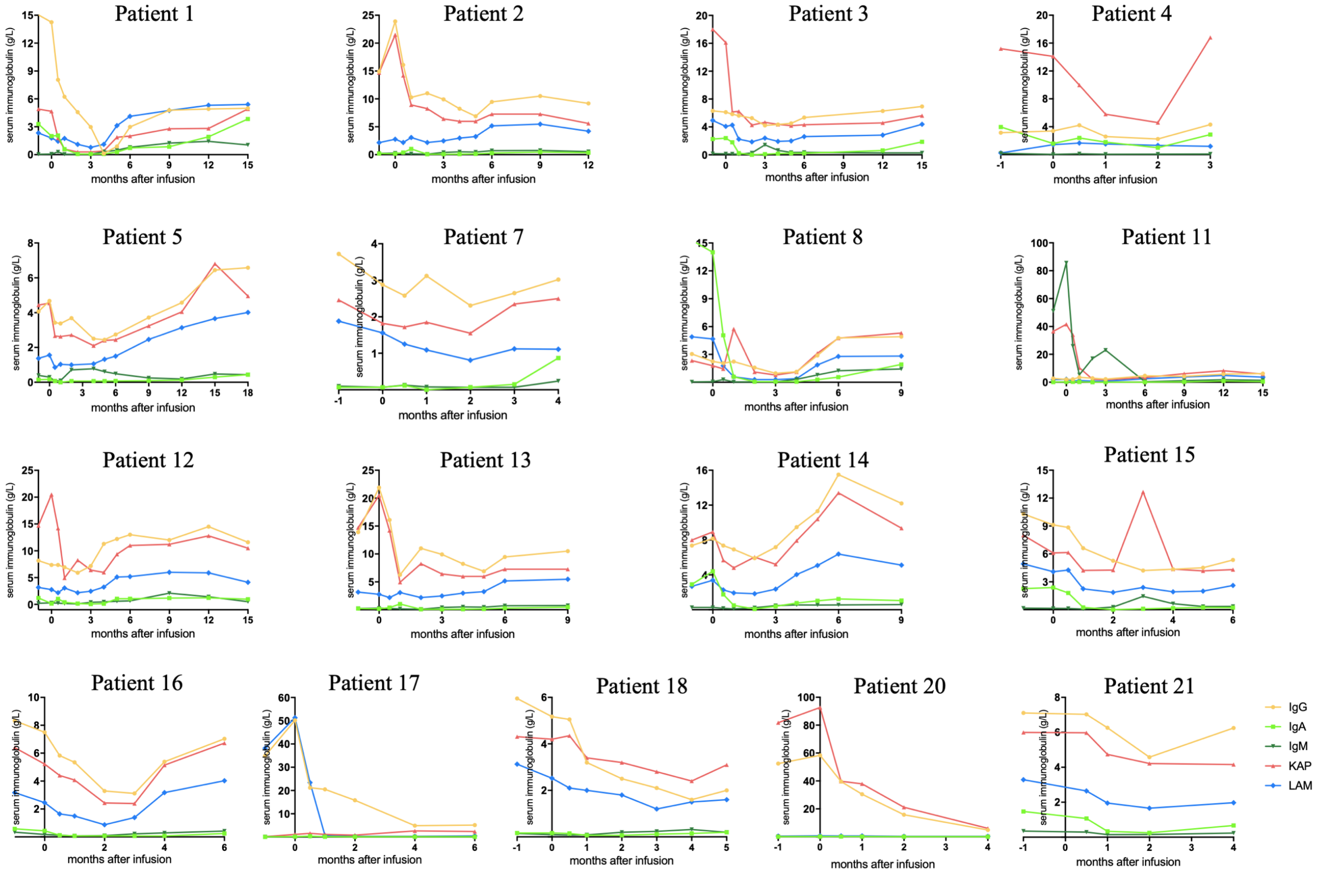

Additional file 1: Figure S5. Immunoglobulin levels following BM38 infusion in patients for over 3 months of follow-up. Normal range of serum immunoglobulin(Ig) is as follows: IgG 7.0-16.0 g/L; IgA 0.7-4.0 g/L; IgM 0.5-2.2 g/L; kappa chain 6.0-13.3 g/L; lambda chain 2.8-6.7 g/L.

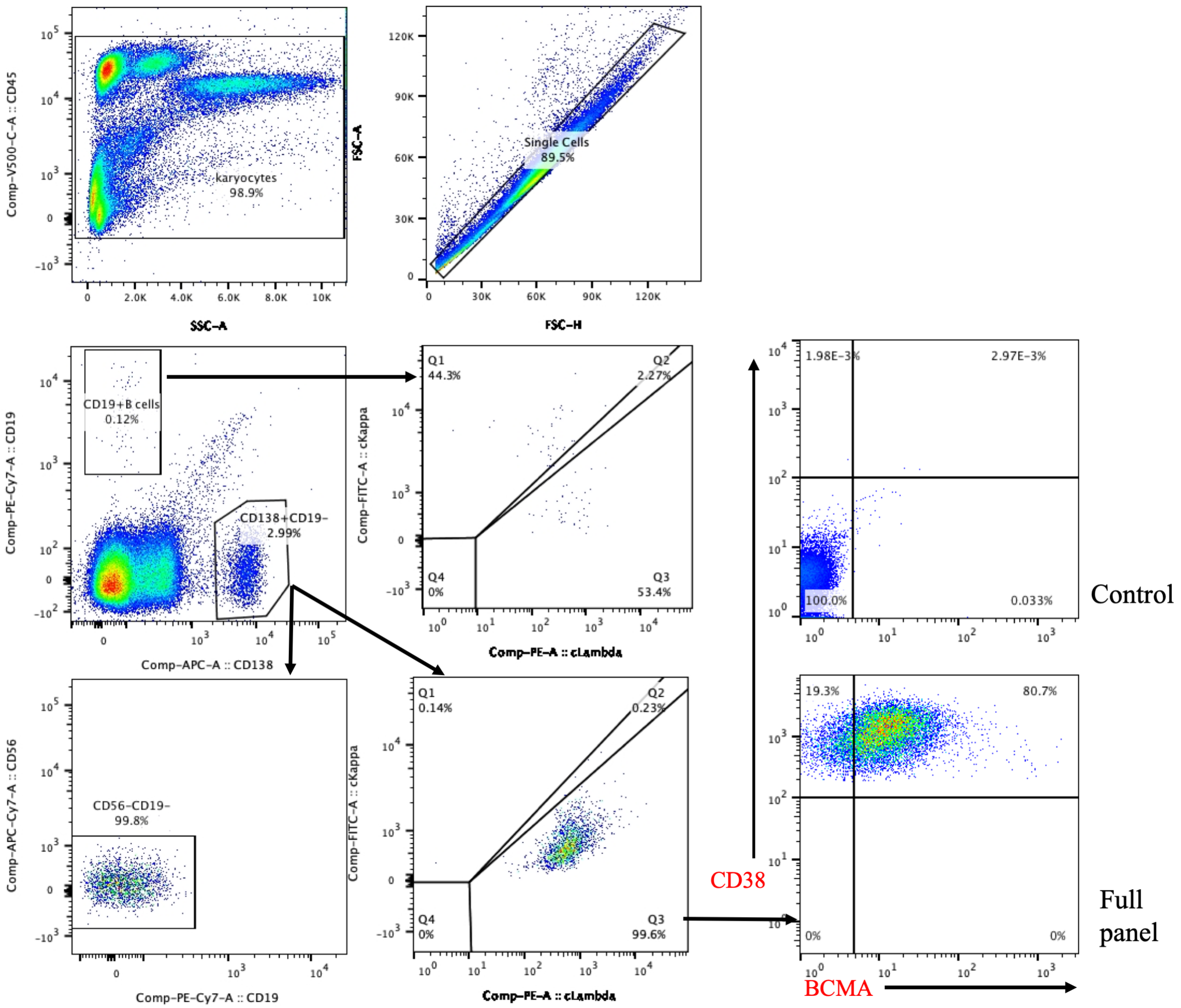

Additional file 1: Figure S6. Representative gating for MM cells and BCMA/CD38 staining. Bone marrow aspirate cells were gated by forward and side scatter, then by singlets. MM cells were identified by gating firstly on CD138^{+}CD19^{-} cells, and then gating on clonal plasma cells using CD56 and kappa/lambda staining. Staining is shown for bone marrow samples from patient 13, and MM cells were CD138^{+}CD19^{-}CD56^{-}lambda+. The % BCMA+ and CD38+ were determined using the control without staining of anti-BCMA-PE and anti-CD38-APC antibodies.

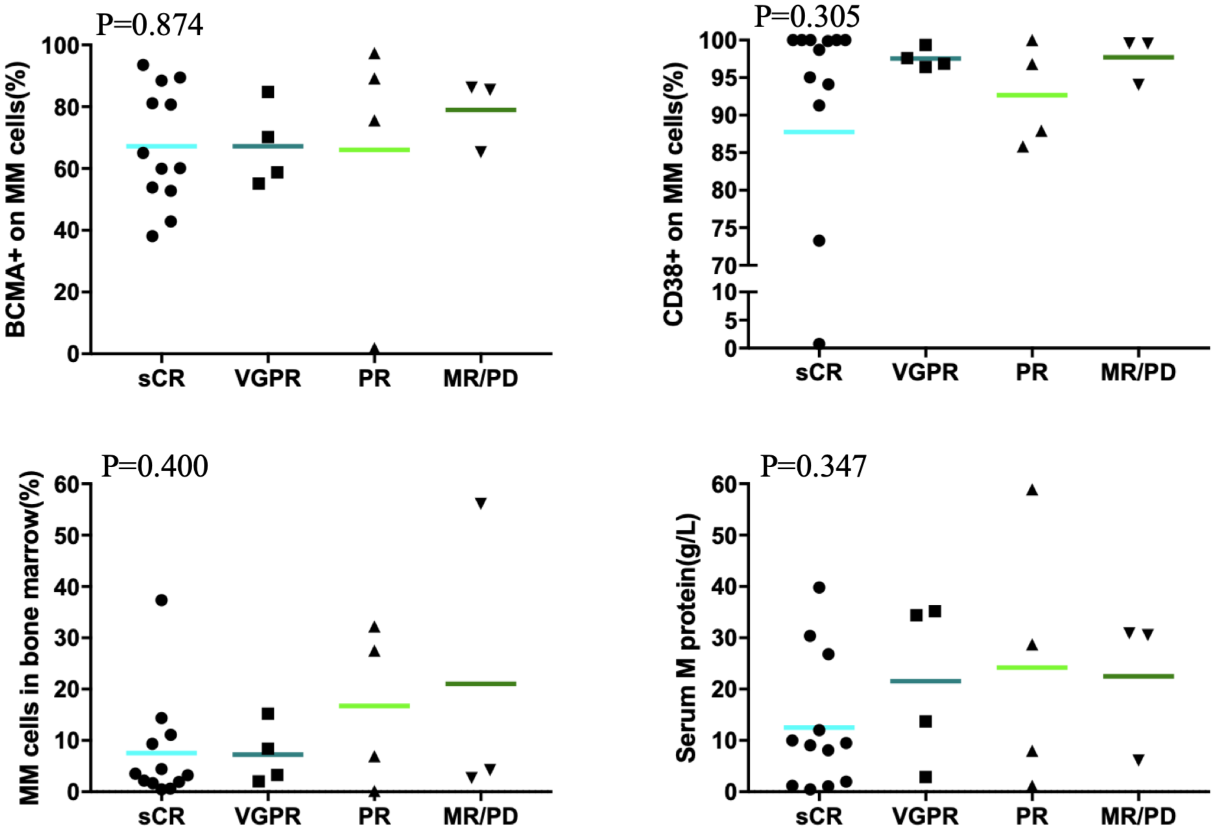

Additional file 1: Figure S7. Correlation analysis of responses. BCMA expression, CD38 expression, MM cells in bone marrow, and serum M protein levels were independent of clinical responses. One-way ANOVA was utilized.

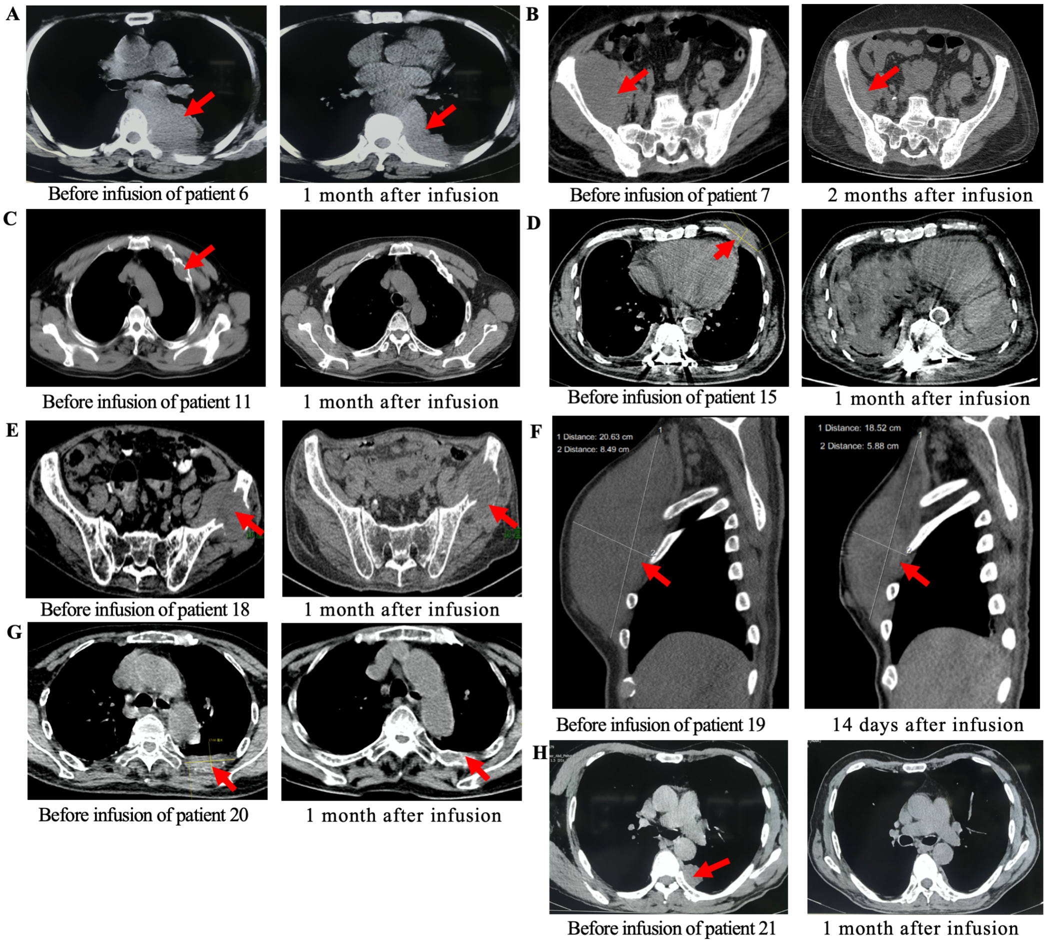

Additional file 1: Figure S8. Responses on extramedullary plasmacytoma. a Patient 6 with a left paraspinal plasmacytoma achieved a PR in month 1. **b** Patient 7 with a right parailiac lesion achieved a VGPR in month 2. **c** Patient 11 with a parathoracic lesion achieved a sCR in month 1. **d** Patient 15 had a lesion in the left chest wall, which was completely eradicated in month 1. **e** Patient 18 with a left parailiac lesion achieved a PR in month 1. **f** Patient 19 had a lesion sized as 20.63 x 8.49cm in the right anterior chest wall, whose condition deteriorated on day 14 after infusion and the lesion was measured as 18.52 x 5.88cm. **g** Patient 20 had a left paraspinal lesion, who achieved a PR in month 1 whereas his extramedullary lesion completely disappeared. **h** Patient 21 had coexistence of 5 cytogenetic profiles and a left pleural plasmacytoma, which disappeared in month 1.

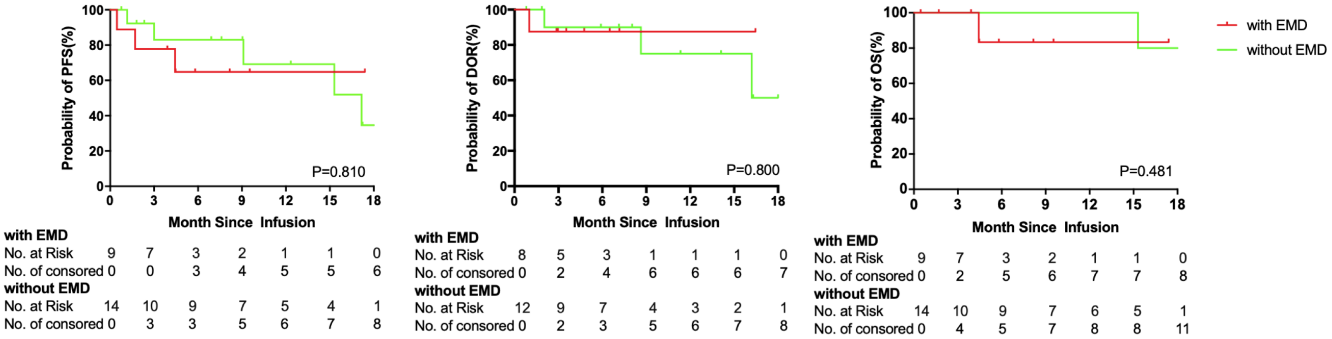

Additional file 1: Figure S9. Kaplan-Meier survival plots of patients according to EMD. The log-rank test was used for comparison. PFS: progression-free survival; DOR: duration of response; OS: overall survival.

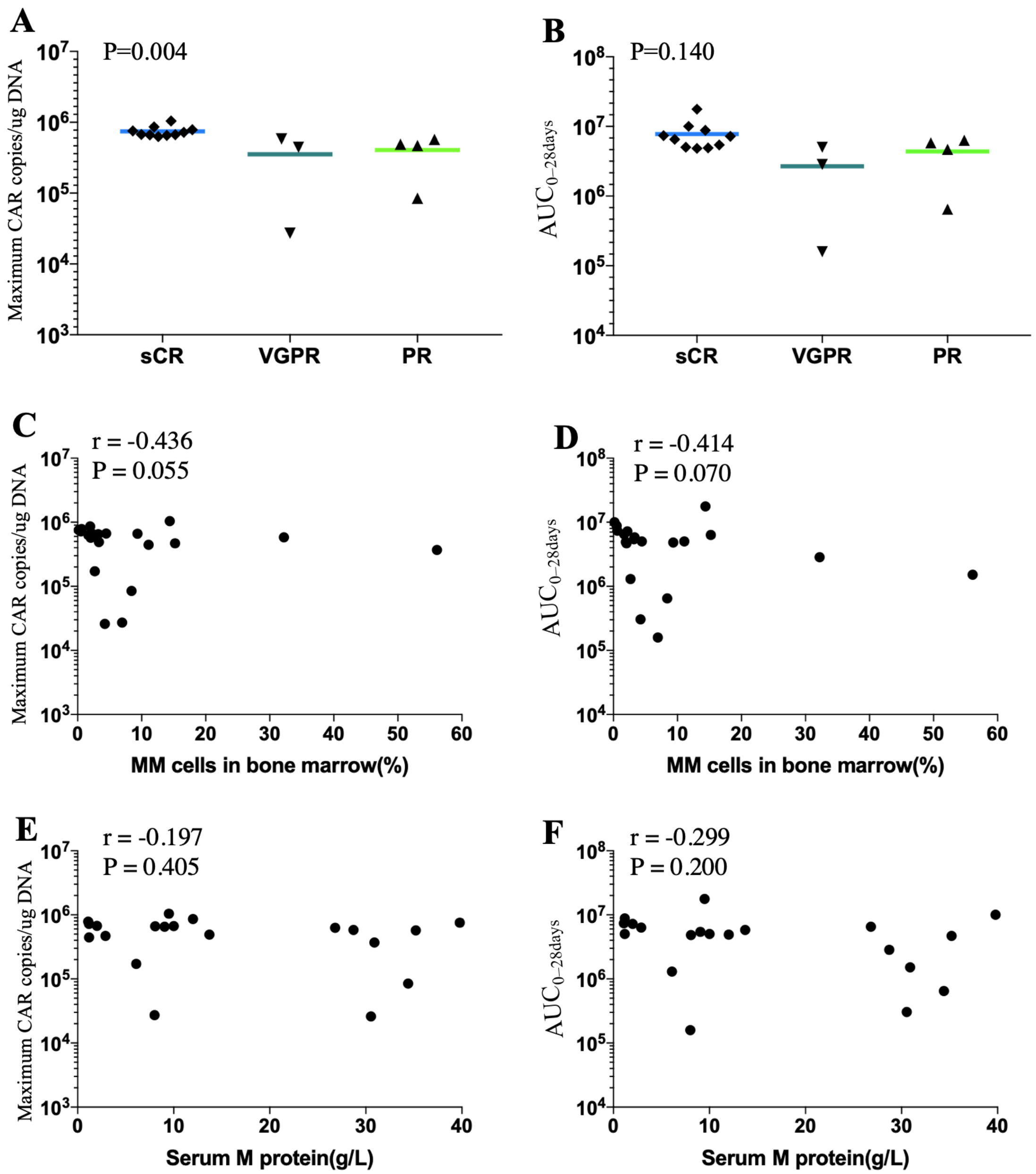

Additional file 1: Figure S10. Correlation analysis of the *in vivo* BM38 expansion. a,b The peak expansion and $AUC_{0\text{-}28days}$ of BM38 CAR-Ts in patients with different responses. One-way ANOVA was used. **c,d** Spearman correlation analysis between MM cells in bone marrow and the peak BM38 expansion and $AUC_{0\text{-}28days}$. **e,f** Spearman correlation analysis between serum M protein levels and the peak BM38 expansion and $AUC_{0\text{-}28days.}$

Table S1. Baseline Characteristics of Individual Patients

ID	Gender	Age	Monoclonal type	Diagonstic time(month)	R-ISS stage	Prior treatment	MM burden at baseline					IHC for EMD#	
							Serum M protein(g/L)	MM cells in BM	BCMA+	CD38+		BCMA	CD38
1	male	56	IgG-KAP	24	Ⅲ	9	12	1.95%	53.91%	98.71%			
2	female	50	IgG-KAP	18	Ⅲ	3	34.41	8.40%	55.10%	97.60%			
3	female	62	KAP	5	Ⅲ	2	8.00	6.96%	97.40%	100.00%			
4	female	70	KAP	96	III	6	8.08	9.34%	52.80%	100.00%			
5	female	63	KAP	8	III	4	2.00	2.17%	93.51%	100.00%			
6	male	55	lgD-LAM	7	II	3	2.90	15.23%	70.18%	99.36%		+	+
7	female	66	IgG-LAM	14	II	3	28.73	32.21%	75.66%	87.95%		+	+
8	female	49	IgA-LAM	40	I	4	9.50	14.39%	65.01%	100.00%			
9	male	72	LAM	24	III	2	6.10	2.68%	65.28%	99.52%			
10	male	50	LAM	48	III	3	30.54	4.26%	85.42%	99.56%			
11	male	57	LAM	36	III	5	9.06	3.22%	42.91%	73.28%		part+	+
12	female	60	IgA-LAM	60	III	9	26.8	1.68%	88.45%	99.89%			
13	female	59	IgG-KAP	14	I	2	10.00	4.44%	80.70%	100%		+	+
14	female	49	IgA-KAP	7	I	2	1.10	0.62%	81.12%	100.00%			
15	female	54	IgA-LAM	19	II	2	1.20	0.5%	59.99%	0.77%		part+	+
16	male	55	IgD-LAM	12	I	4	39.80	11.09%	38.15%	91.30%			
17	male	64	IgA-KAP	12	I	2	13.72	3.31%	84.82%	96.43%			
18	male	63	IgG-KAP	41	II	2	35.20	2.04%	58.82%	96.86%		+	+
19	female	55	IgA-KAP	24	III	2	30.90	56.13%	86.22%	94.05%		+	+
20	male	71	IgG-KAP	161	II	7	1.20	0.17%	1.85%	85.85%		part+	+
21	male	62	LAM	91	II	4	0.50	3.56%	60.11%	95.03%		+	+
22	male	60	IgM-KAP	17	I	3	30.40	37.37%	89.47%	94.12%			
23	female	57	IgG-LAM	15	III	4	58.90	27.50%	89.23%	96.82%			

* BCMA and CD38 expression was defined as the percentage of positive cells in CD45+CD138+CD19- cells.
# IHC was uesd to detect BCMA or CD38 expression on EMD. Part+: less than 50% of abnormal plasma cells express BCMA.
BM: bone marrow; IHC: immunohistochemistry; EMD: extramedullary diseases.

Table S2. Factors possibly associated with severe CRS

Characteristics	Grade 0-2	Grade 3-4	P value
Case, n	18	5	/
MM cells in bone marrow	7.14%	24.14%	P=0.015
Serum M protein at baseline(g/L)	15.25	25.33	P=0.220
Dose of infused CAR-Ts			P=0.618
<4.0×10^{6} BM38 CAR-Ts/kg	7	3	
4.0×10^{6} BM38 CAR-Ts/kg	11	2	
Peak serum CRP(mg/L)	47.09	82.27	P=0.121
Peak serum IL-6 (pg/ml)	166.7	2610	P=0.008
Peak serum ferrin (ng/ml)	8214	33424	P=0.050
Maximum CAR copies/ug DNA	513448	449300	P=0.699
$AUC_{0\text{-}28\ days}$	5738796	4723406	P=0.737

CRP: C-reactive protein; IL-6: interleukin-6; AUC0-28 days: area under the curve of CAR copies per microgram of genomic DNA in the first 28 days after infusion.
Unpaired t test was used for continuous variables, and the fisher's exact test was used for categorical variables.

Table S3. Response and CRS According to Dosage of BM38 CAR-Ts.

Dosage (BM38 CAR+T cells/kg)	0.5×10^6–3.0×10^6 (n=10)	4.0×10^6 (n=13)	0.5×10^6–4.0×10^6 (n=23)
Objective Response			
No. of responders	8	12	20
Rate (95% CI)	80 (62-98)	92 (44-98)	87 (66-97)
Best Overall Response<br>No. and rate (%)			
Stringent Complete Response	4 (40)	8 (62)	12 (52)
Very Good Partial Response	2 (20)	2 (15)	4 (17)
Partial Response	2 (20)	2 (15)	4 (17)
Minor Response	1 (10)	0	1 (4)
Stable/Progressive Disease	1 (10)	1 (8)	2 (9)
Negativity for MRD			
No. and rate (%)	8 (80)	12 (92)	20 (87)
Long-term Outcomes			
Median PFS (month)	9.1	NR*	17.2
1-year DOR (%)	60.0	100	76.4
1-year OS (%)	83.3	100	93.3
Cytokine Releasing Syndrome per Lee criteria<br>No. and rate (%)			
All grade(Grade ≥1)	8 (80)	12 (92)	20 (87)
Severe (Grade ≥3)	3 (30)	2 (15)	5 (22)
Cytokine Releasing Syndrome per ASTCT Consensus<br>No. and rate (%)			
All grade(Grade ≥1)	8 (80)	12 (92)	20 (87)
Severe (Grade ≥3)	2 (20)	2 (15)	4 (17)

The fisher's exact test was used for categorical variables. DOR, PFS, and OS of patients were determined by using the Kaplan-Meier method and compared by utilizing the log-rank test. *P=0.022.

Table S4. Information of infused BM38 CAR-Ts.

ID	Fraction of CAR+/CD3+ T cells(%)	Dosage of infused BM38(cells/kg)	Total number of infused BM38 cells	Times of infusions	Expansion folds on day5
1	30.1%	0.5×10^6	2.95×10^7	1	50.2
2	14.2%	0.5×10^6	3.00×10^7	1	21.2
3	18.9%	1.0×10^6	4.30×10^7	1	30.2
4	27.2%	1.0×10^6	5.70×10^7	1	28.6
5	32.1%	2.0×10^6	1.08×10^8	1	72.9
6	24.6%	2.0×10^6	1.20×10^8	1	24.0
7	20.4%	2.0×10^6	1.16×10^8	1	32.4
8	35.0%	3.0×10^6	1.38×10^8	1	47.3
9	30.0%	3.0×10^6	1.73×10^8	1	29.0
10	30.1%	3.0×10^6	1.65×10^8	1	11.4
11	36.1%	4.0×10^6	2.40×10^8	2	62.8
12	35.0%	4.0×10^6	2.08×10^8	2	66.2
13	49.6%	4.0×10^6	2.60×10^8	2	14.5
14	41.6%	4.0×10^6	2.48×10^8	2	33.6
15	40.0%	4.0×10^6	2.52×10^8	2	25.5
16	38.4%	4.0×10^6	2.32×10^8	2	30.4
17	15.7%	4.0×10^6	3.04×10^8	2	26.8
18	24.3%	4.0×10^6	2.84×10^8	2	31.2
19	15.0%	4.0×10^6	2.20×10^8	2	43.0
20	60.0%	4.0×10^6	2.40×10^8	2	26.3
21	20.3%	4.0×10^6	2.68×10^8	2	12.4
22	35.0%	4.0×10^6	2.96×10^8	2	13.1
23	12.0%	4.0×10^6	2.68×10^8	2	12.1
